# Role of Dopamine in the Heart in Health and Disease

**DOI:** 10.3390/ijms24055042

**Published:** 2023-03-06

**Authors:** Joachim Neumann, Britt Hofmann, Stefan Dhein, Ulrich Gergs

**Affiliations:** 1 Institut für Pharmakologie und Toxikologie, Medizinische Fakultät, Martin-Luther-Universität Halle-Wittenberg, 06097 Halle, Germany; 2 Herzchirurgie, Medizinische Fakultät, Martin-Luther-Universität Halle-Wittenberg, 06097 Halle, Germany; 3 Medizinische Fakultät, Rudolf-Boehm-Institut für Pharmakologie und Toxikologie, Universität Leipzig, 04107 Leipzig, Germany

**Keywords:** dopamine, dopamine receptors, dopamine metabolism, human heart

## Abstract

Dopamine has effects on the mammalian heart. These effects can include an increase in the force of contraction, and an elevation of the beating rate and the constriction of coronary arteries. Depending on the species studied, positive inotropic effects were strong, very modest, or absent, or even negative inotropic effects occurred. We can discern five dopamine receptors. In addition, the signal transduction by dopamine receptors and the regulation of the expression of cardiac dopamine receptors will be of interest to us, because this might be a tempting area of drug development. Dopamine acts in a species-dependent fashion on these cardiac dopamine receptors, but also on cardiac adrenergic receptors. We will discuss the utility of drugs that are currently available as tools to understand cardiac dopamine receptors. The molecule dopamine itself is present in the mammalian heart. Therefore, cardiac dopamine might act as an autocrine or paracrine compound in the mammalian heart. Dopamine itself might cause cardiac diseases. Moreover, the cardiac function of dopamine and the expression of dopamine receptors in the heart can be altered in diseases such as sepsis. Various drugs for cardiac and non-cardiac diseases are currently in the clinic that are, at least in part, agonists or antagonists at dopamine receptors. We define the research needs in order to understand dopamine receptors in the heart better. All in all, an update on the role of dopamine receptors in the human heart appears to be clinically relevant, and is thus presented here.

## 1. Introduction

**Figure 1:**
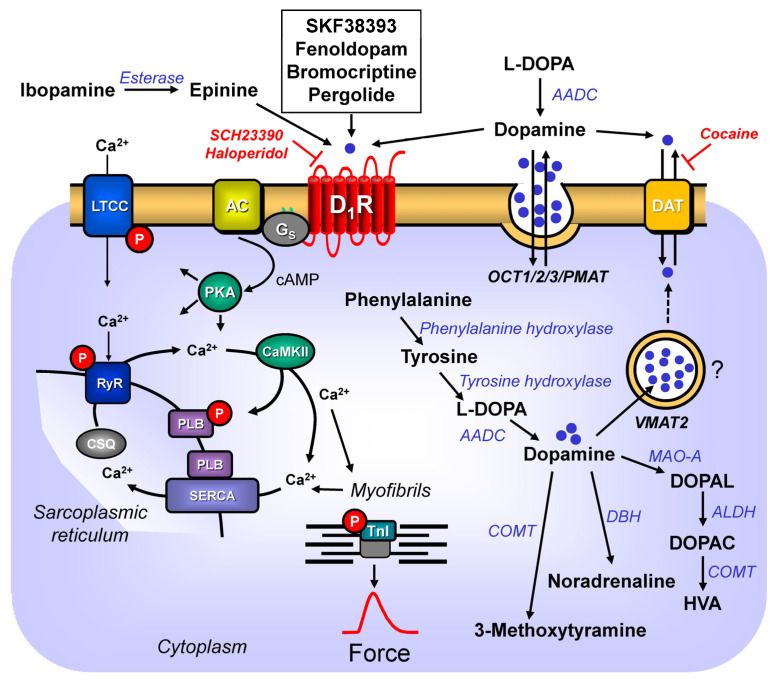
Dopamine might stimulate D_1_-dopamine receptors in the sarcolemma. This would activate the stimulatory GTP-binding protein (Gs), and thereby, the activity of adenylyl cyclases (AC) would be enhanced. AC will catalyze the conversion of ATP to cAMP. This cAMP can activate cAMP-dependent protein kinases (PKA), leading to an increase in the phosphorylation states of regulatory proteins. For instance, phosphorylation of L-type Ca^2+^ channels (LTCC) will lead to more of an influx of Ca^2+^ from the extracellular space. This Ca^2+^ will facilitate the release of Ca^2+^ through the phosphorylated ryanodine receptor (RYR) into the cytosol. The cytosolic Ca^2+^ activates a Ca^2+^ calmodulin-dependent protein kinase (CaMKII) that can phosphorylate phospholamban (PLB), the inhibitory protein of the sarcoplasmic reticulum Ca^2+^ ATPase (SERCA). Possibly, dopamine can also be synthesized in cardiomyocytes. Phenylalanine is oxidized by phenylalanine hydroxylase to tyrosine. Tyrosine is in due course oxidized by tyrosine hydroxylase to L-DOPA, and aromatic L-amino acid decarboxylase (AADC) forms dopamine. Dopamine can be oxidized by dopamine-β-hydroxylase (DBH) to noradrenaline. Catechol-*O*-methyl transferase (COMT) forms 3-methoxytyramine. Monoamine oxidase A (MAO-A) will oxidize dopamine to dihydroxyphenylacetaldehyde (DOPAL) and aldehyde dehydrogenase (ALDH) to dihydroxyphenylacetic acid (DOPAC), and finally, COMT will form homovanillic acid (HVA). Dopamine, at least in nerve cells, can be transported by a vesicular monoamine transporter (VMAT2) into vesicles. These vesicles can release dopamine to the extracellular space. Dopamine can enter the cell via a dopamine transporter (DAT) or via other transporters (OCT, organic cation transporter; PMAT, plasma membrane monoamine transporter). Additionally, the D_1_-dopamine receptor can also be stimulated (see Table 1A) by fenoldopam, SKF38393, bromocriptine, pergolide, and possibly epinine. Epinine is formed from ibopamine through the activity of esterases. The D_1_-dopamine receptor will be blocked by haloperidol, but also by SCH23390 (see Table 1B).

Dopamine is a catecholamine (Figure 1). Dopamine can be of exogenous or endogenous origin. Dopamine has been studied for decades, and a wealth of information on dopamine has been accumulated in the literature. Dopamine has many functions of the mammalian body in health and disease. Practically all organs contain dopamine and can synthesize dopamine. Hence, dopamine must subserve a very critical role in all animals, including humans. Most efforts have been put into the role of dopamine in the central nervous system. In the brain, dopamine can alter the mood of the person. Dopamine depletion in the brain occurs when Morbus Parkinson develops. A lot of work has been focused on how to alter this reduction in dopamine in Morbus Parkinson. Dopamine plays another important role(s) in psychiatric diseases: many antipsychotic drugs (neuroleptic drugs) interfere with dopamine action in the brain. Perhaps it is worth mentioning the many side effects of recreational drugs such as lysergic acid diethylamide (LSD) that may involve dopamine receptors in the brain.

However, in the peripheral organs, dopamine is also found, and in them, dopamine might be of therapeutic and pathological relevance. In this respect, we will mainly focus our attention into the heart and the circulation. In the context of cardiac function and circulation, the role of dopamine in the kidney is important, and it will also be mentioned in the present review.

Dopamine has direct effects on the mammalian heart. These effects can include an increase in the force of contraction, an elevation of the beating rate, and the constriction of the coronary arteries. Depending on the species studied, positive inotropic effects were strong, very modest, or absent, or even negative inotropic effects occurred. We will first start with the formation and degradation of dopamine, we will then move on to dopamine receptors and switch dopamine in cardiovascular pathology, ending with options to use dopamine receptors for cardiac drug therapy. We will always compare animal and human data, stressing on the clinical relevance of the findings. 

## 2. Dopamine Synthesis

Dopamine (3,4-dihydroxyphenethylamine or 3-hydroxytyramine) has been called the third endogenous catecholamine, next to adrenaline and noradrenaline (e.g., [16,17]). Dopamine (earlier, also named oxytyramin [18]) is an important endogenous biogenic amine in mammals. Dopamine is thought to play some role in diseases such as schizophrenia and Morbus Parkinson (review: [19]). Dopamine has been known since the 1950s to be a neurotransmitter in the central nervous system. In this regard, it is important to keep in mind that dopamine does not pass the blood–brain barrier [20]. Dopamine found in the heart or plasma is thus produced in peripheral organs [20]. Indeed, outside the brain, dopamine is mainly produced in the cortex of the adrenal gland, and in the gut [20]. In the plasma, low levels of dopamine were detected (0.1–0.2 nM), that were suggested to originate from nerve cells [21]. In brief, dopamine is built in the human body from the amino acid L-tyrosine (Figure 1) via at least two pathways. L-tyrosine (L-4-hydroxyphenylalanine) itself is formed by hydroxylation in the body from L-phenylalanine, an essential amino acid. A phenylalanine-hydroxylase converts L-phenylalanine to L-tyrosine. In youth, phenylalanine-hydroxylase is solely found in the kidney and liver of mammals [22]. However, upon ageing, phenylalanine-hydroxylase is expressed also in cardiomyocytes (mouse, man: [22]). Hence, over time, the cardiac production of tyrosine as the precursor of dopamine will increase. The following minor pathway for dopamine generation is known, at least in the brain, and potentially in the heart: L-tyrosine can be first decarboxylated by a decarboxylase to tyramine (4-hydroxyphenyl-ethylamine), and then tyramine is hydroxylated by cytochrome 2D6 [23]. Interestingly, cytochrome 2D6 is also expressed in the human heart [24]. Hence, dopamine might also be formed from tyramine in the human heart. This cardiac pathway has apparently not yet been reported in humans. One would predict that medicinal drugs that inhibit the activity of cytochrome 2D6 (review of inhibitory drugs: [25]) should, in part, reduce the generation, and thus the levels of dopamine in the heart, as they would inhibit this alternative pathway. In the main pathway (Figure 1), tyrosine is converted by the enzyme tyrosine-hydroxylase (Figure 1) to DOPA (L-3,4-dihydroxyphenylalamine, L-DOPA, Levo-DOPA). This hydroxylation is the rate-limiting step in the dopamine-synthesis (review: [20]). This tyrosine-hydroxylase is present in the heart [26,27]. Tyrosine-hydroxylase is phosphorylated, and thereby activated by means of a cAMP-dependent protein kinase [28]. Isoprenaline, a β-adrenoceptor agonist and thus a cAMP-increasing agent, increased the phosphorylation state of tyrosine-hydroxylase in isolated rat ganglia [29]. Thus, it would be informative to study whether isoprenaline might increase the phosphorylation state of tyrosine-hydroxylase in the human heart. This has apparently not yet been reported. Should that be the case, there might be a way for how β-adrenergic stimulation could rapidly increase dopamine levels and thus amplify the role of dopamine in the heart. In principle, the activity of tyrosine-hydroxylase can vary regionally in the heart: an increased activity of tyrosine-hydroxylase was noted in the canine sinus node, compared to the canine working myocardium. This might indicate an important, but still not understood, role of dopamine for the initiation of the heartbeat [30,31]. After the constitutive deletion of tyrosine-hydroxylase in mouse, less DOPA and dopamine were formed in the body, as would be expected for a rate-limiting enzyme [32]. When tyrosine-hydroxylase was knocked down selectively in the sympathorenal system, the dopamine levels and the noradrenaline levels in the mouse heart decreased [33]. This indicates that some of the dopamine found in the heart originates from sympathetic neurons [33]. DOPA loses its acid structure via the activity of a decarboxylase (Figure 1), and thus, dopamine is formed [31]. The enzyme decarboxylase is also found in the heart [31]. Hence, the decarboxylation from dopamine might take place in the heart.

## 3. Dopamine Levels and Metabolism in the Heart

Convincing data support the presence of dopamine in the mammalian heart. These include the canine left ventricle (40.9 ng/g; 0.24 µM [34]), in the heart of rats (468 ng/g; 2.81 µM, [35]), or in the septum of the human heart (100 ng/g: 0.67 µM). Even after orthotopic cardiac transplantation, dopamine was detected, albeit in lower concentrations, in the human heart (25 ng/g: 0.17 µM [36]). In mouse heart, one reported similar dopamine concentrations, namely, 0.7 pg/mg protein (0.47 µM [33]). In the rat atrium, about 20 ng/g of dopamine, in contrast to dopamine levels of about 1 ng/g, in the rat ventricle were reported, indicating a regional difference [37]. These concentrations of dopamine are, at least in the organ bath, high enough to exert positive inotropic effects in isolated human cardiac preparations [38]. Thus, dopamine concentrations present in the heart might be relevant to sustaining cardiac contractility in humans. As mentioned before, all dopamine-synthetizing enzymes are present in the heart, and possibly even in cardiomyocytes. Therefore, it is conceivable that dopamine is not only present in the heart, but even produced in cardiomyocytes [20]. However, this issue needs further experimental studies. Any role of dopamine within the cardiomyocyte is speculative: dopamine might be a metabolic intermediate or a pre-drug for noradrenaline (Figure 1); possibly, dopamine serves autocrine and paracrine purposes: dopamine might stimulate dopamine receptors on cells such as cardiomyocytes, cardiac endothelial cells, or cardiac smooth muscle cells [39].

How is dopamine then degraded? Dopamine can be converted by a dopamine-β-hydroxylase that is also expressed in the heart [26] to noradrenaline (Figure 1). In this way, dopamine would act as a pro-drug of noradrenaline. When one constitutively deleted dopamine-β-hydroxylase, no noradrenaline was formed, indicating that dopamine-β-hydroxylase is the rate-limiting enzyme for the formation of noradrenaline in general [40]. It would be interesting to know how the noradrenaline level in the heart would change if the heart- or even cardiomyocyte-specific deletion of dopamine-β-hydroxylase were performed. If the cardiac production of noradrenaline from dopamine were relevant, one might predict then that little noradrenaline would be present in the heart, but normal levels of noradrenaline in all other tissue. Moreover, dopamine is degraded by means of catecholamine-O-methyltransferase (COMT) (Figure 1) to 3-methoxytyramine. In addition, dopamine in the mitochondria is oxidized by means of monoamine oxidases (MAO), and thus, it can also be converted to (Figure 1) 3-methoxytyramine [20]: consistent with this scheme, after the genetic deletion of COMT or MAO, the dopamine levels in the organs of mice increased [41,42].

Extracellular dopamine can reach the cytosol of a cell via a dopamine-transporter (DAT) (Figure 1), which is also found in the heart [43]. At least within the cell bodies of nerve cells, noradrenaline and dopamine can be transported by a vesicular monoamine-transporter (VMAT2) (Figure 1) into storage vesicles, where dopamine is protected from enzymes that might degrade dopamine [20,43]. In addition, this dopamine-transporter (VMAT2) has been detected in mammalian hearts [43]. A role of VMAT2 in cardiomyocytes needs to be better defined: cardiomyocytes do not contain storage vesicles for monoamines. Therefore, it might be worthwhile to study whether or not VMAT2 is expressed on the mRNA- or on the protein-level in cardiomyocytes. In the kidney, sufficient dopamine is produced locally that these renally formed dopamine levels are high enough to stimulate renal dopamine receptors. Indeed, hypertension might result in some patients from their inability to produce enough dopamine in their kidneys (review [44]). We are tempted to speculate that such defects might also be present in the human heart.

## 4. Dopamine and D_1_-Dopamine Receptors, Especially in the Animal Heart: Signal Transduction and Regulation

### 4.1. General Dopamine Receptor Classification

Dopamine (Table 1A,B) might act in the heart through five known dopamine receptors (D_1_-, D_2_-, D_3_-, D_4_-, D_5_-dopamine- receptors, Table 1) but dopamine is also an agonist at the cardiac α- and β-adrenoceptors (Table 2 and Table 3), and for other receptors or proteins such as transporters (Table 4). D_1_-dopamine- and D_5_-dopamine receptors, which may be regarded as one family (presenting no exons: “D_1_-like”), have in common that they both stimulate the enzymatic activity of adenylyl cyclases. This holds also true in the heart: In plasma membranes from rat hearts, dopamine and the D_1,5_-dopamine receptor agonist SKF 38393 (Table 1A) increased the activity of adenylyl cyclases in a concentration-dependent fashion: this effect could be blocked by the D_1,5_-dopamine receptor antagonist SCH 23390 (Table 1B), suggesting that the D_1,5_-dopamine receptor couples to cAMP-formation in the heart [45]. Earlier work in isolated perfused rat heart revealed time- and concentration-dependent increases of cAMP content by dopamine (1 µM dopamine and higher [46]. However, it is likely that this increase in cAMP in the rat heart was mainly mediated by β-adrenoceptors, as these cAMP-increasing effects are antagonized by β-adrenoceptor antagonists. Moreover, in brain cells, the stimulation of D_1_-dopamine receptors via cAMP-dependent protein kinase can activate or inhibit diverse ion channels [47]. In the heart, the stimulation of the D_1_-dopamine receptor will activate ion channels (see Section 5.8). D_1_-dopamine receptors might also activate (or inhibit), directly (without cAMP) via the βγ-subunits of guanosine triphosphate-binding proteins, some ion channels [47]. 

The D_1_-dopamine receptor resides on human chromosome 5q35.1 and encodes a 446 amino acid protein [44]. Polymorphisms of the D_1_-dopamine receptor gene have been reported and linked to hypertension [44]. Usually, as mentioned above, dopamine acting on the D_1_-dopamine receptors can activate canonical pathways (cAMP increase) as well as non-canonical pathways (β-arrestin—MAPK-kinase). Mass drug screening efforts detected structurally dissimilar compounds (but still dopamine receptor agonists) that use mainly the canonical pathway instead of the non-canonical pathway of the D_1_-dopamine receptor [74]. These compounds also induced less desensitization of the D_1_-dopamine receptors (see Section 4.8) than dopamine itself, leading to the hypothesis of clinically usable biased agonism [74]. Furthermore, at least in the brain, the stimulation of D_1_-dopamine receptors led to phosphorylation of c-AMP responsive element binding protein (CREB) [75]. Such a CREB phosphorylation was reported upon isoprenaline addition in the rat heart (e.g., [76]). It has apparently not been reported whether dopamine in the heart can likewise lead to CREB phosphorylation, and which receptor might be involved.

In the rat heart, there are D_1_-dopamine receptors on cardiomyocytes, endothelial cells, and smooth muscle cells [45]. The density of the D_1_-dopamine receptors was higher in the rat atrium than in the rat ventricle, and it was highest in the sarcolemma [45]. However, the authors also found D_1_-dopamine receptors in the cytosol of rat cardiomyocytes, and speculated that it might have a function within the cardiomyocytes [45]. D_1_-dopamine receptors could also be detected with polymerase chain reaction (PCR) from rat ventricular RNA, and via the in situ amplification of mRNA in slices from the rat heart [45]. In the rat atrium (left and right), the expression of the mRNA for the D_2_-, D_3_-, and D_4_-dopamine receptor has been reported [77], however, it was not reported in which cell type the receptors were located. The D_1_-dopamine receptor was also detected in Western Blots of rat atria and ventricles at about 70 kDa; larger than the calculated size of 50 kDa possibly due to glycosylation and phosphorylation [45]. With antibodies on Western blots, one has identified D_1_-dopamine receptors in the atrium, ventricle, and coronary arteries of adult rats [45], but later also in neonatal rat cardiomyocytes [78]. In the rat atrium (left and right), the expression of the mRNA for the D_2_-, D_3_- and D_4_-dopamine receptor has been reported; [77]. One should regard the findings on mRNA levels as being much more convincing than the data with the antibodies. The antibody specificity of guanosine triphosphate-binding proteins-coupled receptors is a notoriously controversial area. In our view, the best control for these antibodies is through using on the same Western blot or immunohistology a sample from a D_1_-dopamine receptor knock-out heart. To our knowledge, this has not yet been published.

In contrast to the “D_1_-like”-receptors, D_2_-, D_3_-, D_4_-dopamine receptors (no exons) are placed into a different family (“D_2_-like”), and its members have in common that they can inhibit the enzymatic activity of adenylyl cyclases. “D_2_-like”-receptors use other targets than “D_1_-like” dopamine receptors for signal transduction. For instance, “D_2_-like”-receptors can inhibit neuronal Ca^2+^ channels. It is currently unknown, but it might be interesting to study whether “D_2_-like”-dopamine receptors can also inhibit cardiac L-type Ca^2+^ channels. This would reduce cytosolic Ca^2+^ concentrations, and should lead to a negative inotropic effect, or at least diminish a positive inotropic effect that dopamine would exert via the “D_1_-like”-receptors mentioned above. Moreover, in rat neonatal cardiomyocytes, D_2_-dopamine receptor stimulation (by bromocriptine) activated, and in the additional presence of haloperidol, reduced the ERK1/2 pathway and the PI_3_K-Akt-GSK3β pathway [79]. In rat neonatal cardiomyocytes, bromocriptine induced the translocation of the protein kinase C-epsilon into the cell membrane [79]. These data are not as clear-cut as they may seem. Bromocriptine above a concentration of 0.1 µM (Table 1A) will also activate D_1_-dopamine receptors, and haloperidol would also be antagonistic at the D_1_-dopamine receptors (Table 1B). Hence, such experiments should be confirmed using, e.g., more specific agonists or antagonists.

Via pertussis toxin-sensitive guanosine triphosphate-binding proteins, D_4_-dopamine receptors inhibit forskolin-stimulated cAMP generation in a neuronal cell line [80]. Whether this occurs in cardiomyocytes, especially human ventricular cardiomyocytes, has not yet been reported. Here again, our tools are limited as there are no very selective agonist or antagonists that allow us to distinguish functionally between the members of the “D_2_-like”-family of receptors (see Table 1A compared to Table 1B). Here, genetic models would possibly be more helpful.

### 4.2. Signal Transduction of Dopamine Receptors via PP2A

**Figure 2:**
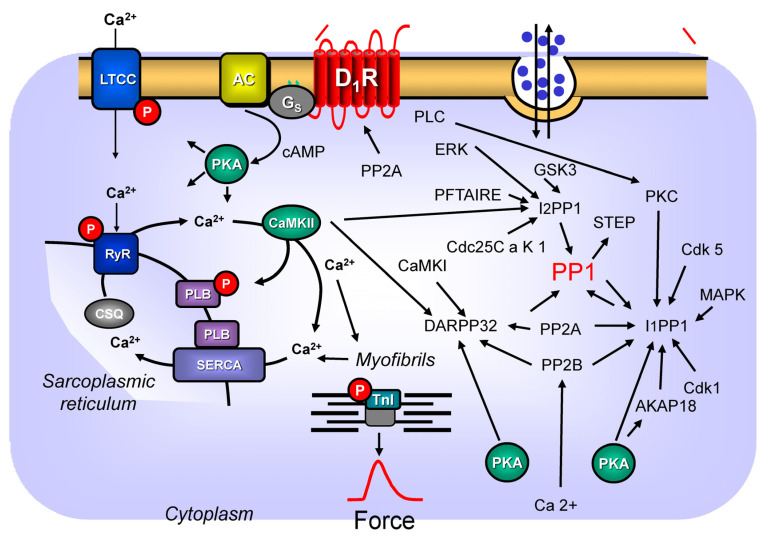
Speculative mechanisms of D_1_-dopamine receptor-mediated signal transduction in the heart. These pathways are known in the central nervous system, but they need to be elucidated in the human heart. Protein phosphorylations can be reversed via the action of protein phosphatases, such as PP1, PP2A, or PP2B. The so-called “dopamine- and cAMP-regulated phosphoproteins with an apparent weight of 32 kDa” (DARPP32), protein phosphatase-1-inhibitor-1 (I-1) protein phosphatase-1-inhibitor-2 (I-2), can inhibit PP1 and thus amplify the function of the D_1_-dopamine receptor. The inhibitory actions of I-1 and DARPP32 on PP1 are amplified after their phosphorylation by cAMP-dependent protein kinase (PKA). I-1 and DAPRP32 are dephosphorylated, and thus they are inactivated by PP2A and PP2B. PP2A might increase the insertion of D_1_-dopamine receptors (D_1_-R) into the sarcolemma. I-2 can be regulated in its activity to inhibit PP1 by the kinases shown here: casein kinase II (CamKII), glycogen synthase kinase 3 (GSK-3), a kinase abbreviated as PFTAIRE kinase (PFTAIRE), by ERK, and a Cdc25C-associated kinase 1 (Cdc25C a K). I-1 is phosphorylated, and its activity is altered by Cdk1, CdK5, protein kinase C (PKC), and a mitogen-activated kinase (MAPK). See text for further details.

The signal transduction of dopamine receptors involves protein phosphorylation (see Section 4.1). The phosphorylations of proteins on amino acids serine and threonine are reversed by protein phosphatases. Most importantly in cardiac tissue (but also in other organs) are the so-called protein phosphatases 1 and 2A (PP1 and PP2A, respectively). At least in the kidney, the catalytic subunit of the protein phosphatase 2A (PP2Ac) is involved in the signal transduction of the D_1_-dopamine receptor. More specifically, the activation of PP2Ac is necessary for the full function of the D_1_-dopamine receptor: activated PP2Ac leads to a translocation of D_1_-dopamine receptors from the cytosol into the cell membrane where it usually exerts its function, namely, where it stimulates the activity of adenylyl cyclases [81]. Only if the D_1_-dopamine receptor is in the cell membrane (sarcolemma) the D_1_-dopamine receptor can function (Figure 2): in this case, the renal D_1_-dopamine receptor can inhibit Na^+^-transport in the kidney, which is finally responsible for lowering blood pressure in mammals. Likewise, phosphorylated and thus inactivated D_1_-dopamine receptors [82] can be dephosphorylated, and thus, can again be activated by PP2Ac [82,83]. It is a research interest to study whether or not PP2A is relevant in this regard in cardiac cells. The findings on a renal role of PP2A are not as clear as one might wish. The findings are based on the action of okadaic acid as a tool to block PP2Ac. However, several other phosphatases are also inhibited by okadaic acid, and the intracellular concentrations of okadaic acid are usually unknown [84,85]. Thus, this topic should be investigated with newer tools and methods. For instance, one might use transgenic mice with heart-specific overexpression of PP2Ac, heart-specific downregulation of PP2Ac, or hearts from animals heterozygotic for a knock-out of PP2Ac (a complete constitutive knock-out of PP2Ac is lethal: [86]).

### 4.3. Dopamine Receptors and DARPP32

At least in the brain, the stimulation of D_1_-dopamine receptors activates cAMP-dependent protein kinase, and cAMP-dependent protein kinase will phosphorylate a so-called dopamine- and cAMP-regulated neuronal phosphoprotein with an apparent weight of 32 kDa (DARPP32 [87,88] Figure 2). After being phosphorylated, DARPP32 is activated, and in this form, it can inhibit the catalytic subunit of an important serine/threonine phosphatase, namely PP1c [84]. Initial reports using protein-derived antibodies against DARPP32 failed to detect DARPP32, at least in the bovine heart [87]. However, it is likely that this immunological detection method was simply too insensitive to measure DARPP32 in cardiac tissue. More recently, DARPP32 was noted to be expressed (on a protein level and mRNA level) in cardiomyocytes [89]. It would be informative to study whether dopamine could stimulate the phosphorylation of DARPP32 in cardiac cells, which has apparently not yet been reported. Of note, data have accumulated that DARPP32 might be regarded as a “coincidence integrator” in the cell. In other words, the phosphorylation and dephosphorylation of DARPP32 occurs at different amino acids. Different kinases and phosphatases use DARPP32 as their target. Thus, DARPP32 is strategically positioned to integrate competing signals from cell surface receptors. DARPP32 is phosphorylated by both casein kinase I and casein kinase II, leading to an increased or decreased ability of DARPP32 to inhibit the enzymatic activity of PP1 ([84] Figure 2). PP1 is not able to terminate its own inhibition via the dephosphorylation of DARPP32. In contrast, DARPP32 is dephosphorylated in vitro by PP2A and another serine/threonine protein phosphatase, PP2B ([84] calcineurin). These dephosphorylations are clearly regulated. These regulations need more studies for the role of PP2A, but they are quite well understood for PP2B. PP2B in vitro and in vivo is activated as a phosphatase via increased cytosolic levels of free Ca^2+^ ([84] Figure 2). Thus, the present thinking is that Ca^2+^-elevating receptors such as the D_1_-dopamine receptor might activate PP2B. PP2B would dephosphorylate DARPP32 and would thus increase the activity of PP1. PP1 would now dephosphorylate and thus activate a tyrosine phosphatase called STEP. This activated STEP would now dephosphorylate and thus inactivate a number of proteins involved in gene transcription [90]. This is one way in which dopamine receptors can alter gene transcription.

### 4.4. Dopamine Receptors and Inhibitor-1 of PP1

A well-studied cardiac analogue of DARPP32 is the so-called phosphatase inhibitor-1 (review: [84] Figure 2). This protein is, in a similar fashion, as DARPP32 phosphorylated by cAMP-dependent protein kinase, and phosphorylated phosphatase-inhibitor-1 will inhibit PP1 phosphatase activity. An isoprenaline-induced increase in the phosphorylation state of phosphatase-inhibitor-1 has been reported in the in hearts of living guinea pigs, isolated perfused guinea pig heart, in isolated guinea pig cardiomyocytes and isolated perfused mouse hearts [91,92,93,94].

Protein phosphatase 1-inhibitor-1 is phosphorylated at amino acid serine 67 by proline-directed kinases, namely Cdk1, Cdk5, and mitogen-activated protein kinase ([95]). This phosphorylation at serine 67 impaired the ability of cAMP-dependent protein kinase to phosphorylate the amino acid threonine 35 and hence to activate the protein [95]. Dephosphorylation was brought about by PP2A and PP2B (Figure 2 [95]), in a very similar fashion as mentioned above for DARPP32. Later, a phosphorylation of protein phosphatase-1 inhibitor-1 at serine 6 by Cdk5 was additionally detected [96]. Like the phosphorylation at serine 67 and also the phosphorylation at serine 6, this rendered protein phosphatase 1-inhibitor-1 less susceptible to phosphorylation, and thus, activation by cAMP-dependent protein kinase [96]. Hence, one has argued that Cdk5 can indirectly activate PP1 [96]. Serine 6 in protein phosphatase 1-inhibitor-1 could be dephosphorylated by PP1, PP2A, and PP2B [96]. A different amino acid, namely serine 65 in protein phosphatase 1-inhibitor-1 can be phosphorylated by protein kinase C: this phosphorylation is reduced if protein phosphatase 1-inhibitor-1 is already phosphorylated at serine 67 [97], probably allowing fine tuning of PP1 activity in the cell. Moreover, protein phosphatase-1-inhibitor-1 can be phosphorylated at threonine 75 by PKC, leading to a reduced inhibition of the activity of PP1, and thus, enhanced PP1 activity and diminished cardiac function [98]. Furthermore, others have described that protein phosphatase-1-inhibitor-1 can bind to the A-kinase anchoring protein 18 (AKAP18) in rat heart [99]. When they hindered cAMP-dependent protein kinase to bind to AKAP18, this led to a diminution of protein phosphatase-1-inhibitor-1 phosphorylation. PP1c also bound to AKAP18 [99]. Under their experimental conditions, only cAMP-dependent protein kinase bound to AKAP18 would reduce PP1 activity [99]. To sum up, it would be worthwhile to study whether or not dopamine can increase the phosphorylation state of protein phosphatase-1-inhibitor-1 in cardiomyocytes, preferably from human hearts.

### 4.5. Dopamine Receptors and Inhibitor-2 of PP1

Another well characterized protein that can inhibit the enzymatic activity of PP1 and that thus might play a role in dopamine receptor-mediated protein phosphorylation is the protein phosphatase-1-inhibitor-2 (Figure 2). The overexpression of protein phosphatase-1-inhibitor-2 in the heart leads to an enhanced phosphorylation of phospholamban (Figure 1) and increased contractility in the heart [100,101]. Protein phosphatase 1-inhibitor-2 is phosphorylated on threonine 72 by glycogen synthase kinase-3 and on serine 86 by casein kinase II (Figure 2 [102]). Protein phosphatase-1-inhibitor-2 can bind to PP1c and thus inactivate PP1c. However, if protein phosphatase-1-inhibitor-2 in this complex is phosphorylated by glycogen synthase kinase-3, then protein phosphatase-1-inhibitor-2 apparently loses its phosphatase inhibitory activity and PP1c is active. The phosphorylation by casein kinase II of protein phosphatase-1-inhibitor-2 is facilitated, if protein phosphatase-1-inhibitor-2 had been phosphorylated previously by glycogen synthase kinase-3. In other words, protein phosphatase-1-inhibitor-2 might also be regarded as a sensor of casein kinase II and glycogen synthase kinase-3 in the heart [103]. As far as we know, the phosphorylation of protein phosphatase-1-inhibitor-2 in cardiomyocytes was never published, and it may be regarded as a research need. Protein phosphatase-1-inhibitor-2 is present in the heart [102]. More recently, protein phosphatase-1-inhibitor-2 was found to be phosphorylated by Cdc25C-associated kinase 1 (Figure 2) in vitro, and this phosphorylated protein phosphatase-1-inhibitor-2 at amino acid threonine 71, which inhibited the enzymatic activity of the PP1c complex bound to protein phosphatase 1-inhibitor-2 further, and inhibited the phosphorylation of threonine 72 of protein phosphatase 1-inhibitor-2 by glycogen synthase kinase 3. Moreover, a PFTAIRE kinase (Figure 2) could phosphorylate protein phosphatase-1-inhibitor-2 at serine 86, which leads to the facilitated phosphorylation of threonine 72 of protein phosphatase 1-inhibitor-2 by casein kinase II [103]. Threonine 72 on protein phosphatase-1-inhibitor-2 can also be phosphorylated by ERK 1 [104]. Thus, protein phosphatase-1-inhibitor-2 can alter D_1_-dopamine receptor-mediated protein phosphorylation and potentially cardiac function, because D_1_-dopamine receptors can activate via the non-canonical pathway ERK1 (Figure 2).

### 4.6. Dopamine Receptors and Inositol Trisphosphate (IP_3_) Levels

As mentioned above, protein kinase C (PKC) can be activated by dopamine receptors. At least in the brain cells and in renal preparations, D_1_-dopamine receptors can also increase via guanosine triphosphate-binding proteins the activity of protein kinase C, and therefore, IP_3_-levels (e.g., [105] Figure 1). In brain cells, the stimulation of D_1_-dopamine receptors can lead independently of adenylyl cyclases or guanosine triphosphate-binding proteins, namely via β-arrestin, to a rise in IP_3_, subsequent augmentation in levels of intracellular Ca^2+^ and a subsequent activation of CaM-kinases, but it can also lead to the phosphorylation of MAP-kinases (such as ERK1,2: [106]). SKF81297 (Table 1A) and SKF83822 (Table 1A) activate only adenylyl cyclases via D_1_-dopamine receptors in a concentration-dependent manner, whereas SKF83959, another D_1_-dopamine receptor agonist, could not increase cAMP-levels in human embryonic kidney cells stably transfected with D_1_- and D_2_-dopamine receptors, but only IP_3_-levels in cells [9]. It might be fruitful to study whether similar differences in the signal transduction hold true for human cardiac dopamine receptors, and whether this observation might be clinically useful for increasing the force of contraction in the failing human heart without any increase in cAMP-levels.

### 4.7. Upregulation, Downregulation, and Desensitization

In an immortalized cardiac cell line (H9c2), treatment with the D_2_-dopamine receptor antagonist haloperidol (Table 1B) led to the upregulation (increase in density) of D_2_-dopamine receptors and the downregulation (decrease in density) of D_1_-dopamine receptors [15]. In contrast, in rat neonatal cardiomyocytes, the D_2_-dopamine receptor agonist bromocriptine (Table 1A) increased, and the D_2_-dopamine receptor antagonist haloperidol decreased the expression of D_2_-dopamine receptors [80]. This apparent opposite regulation of receptor expression might result from the different model systems studied (neonatal cells versus tumor-derived cell). However, one has to keep in mind that haloperidol (Table 1B) and bromocriptine also act on D_1_-dopamine receptors, which complicates the interpretation of these studies (Table 1A, Figure 1). Like for most heptahelical receptors, guanosine triphosphate-binding proteins-coupled receptor kinases (βARKs) lead to the uncoupling and desensitization of dopamine receptors [42,44,107]. The D_1_-dopamine receptors can also be phosphorylated and regulated by PKC [108]. This desensitization, at least in cell culture with HEK293 cells transfected with the rat D_1_-dopamine receptor, requires the initial phosphorylation of the C-terminal tail of the receptor, and subsequently, the phosphorylation on the third intracellular loop of the D_1_-dopamine receptors [109]. In this way, a conformational change of the D_1_-dopamine receptor comes about, that allows arrestin to bind to the third intracellular loop, leading to receptor and effector uncoupling as the first step of desensitization [109]. The D_1_-dopamine receptor can be phosphorylated by at least the cAMP-dependent protein kinase and βARKs [110]. From these findings, one could assume that in the heart, the inhibition of the phosphorylation of the D_1_-dopamine receptor at the appropriate amino acids should hinder homologous desensitization to dopamine [109]. One could even generate transgenic mice where the phosphorylateable amino acids in the D_1_-dopamine receptor are missing, or where the arrestin binding sequence of intracellular loop three is mutated, such that arrestin cannot bind. Under these conditions, the desensitization of the intact heart to dopamine should not occur. If such polymorphisms in the D_1_-dopamine receptor occurred in patients, the patients should not be able to desensitize to dopamine.

### 4.8. Dimerization of Dopamine Receptors

It is now generally accepted that guanosine triphosphate-binding proteins-coupled heptahelical receptors can form heterodimers and homodimers. Moreover, there are many examples where the dimerization of receptors can alter their affinity for receptor agonists and receptor antagonists, and this can lead to alterations in the general pathway of signal transduction through guanosine triphosphate-binding proteins and effector proteins to alternative pathways where the receptors do not couple via their neighboring guanosine triphosphate-binding proteins, but via other proteins such as arrestins. Dopamine receptors fall into this category. For instance, in the normal mouse brain, one noticed heterodimers of D_1_-dopamine receptors and H_3_-histamine receptors. The dimers of D_1_-dopamine and H_3_-histamine receptors coupled in a direct way (bypassing guanosine triphosphate-binding proteins) to MAP-kinase pathways [111]. The heterodimers of D_1_-dopamine receptors and H_3_-histamine receptors have been suggested to be useful drug targets; for instance, in neurological diseases [112]. H_3_-histamine receptors are lacking in cardiomyocytes, but present in cardiac fibroblasts and thus might be relevant in our context [113]. Heterodimers of D_1_-dopamine receptors and D_2_-dopamine receptors have been described with altered signal transduction pathways compared to non-dimeric receptors: it turned out that in cells transfected with both the D_1_-dopamine receptors and the D_2_-dopamine receptors, the stimulation of D_1_-dopamine receptors and D_2_-dopamine receptors by two selective agonists given together or by one agonist (SKF81297), which stimulate D_1_-dopamine receptors and D_2_-dopamine receptors, led to a greater increase in free Ca^2+^ in these cells than the stimulation of the D_1_-dopamine receptors alone [9]. As both D_1_-dopamine receptors and D_2_-dopamine receptors are present in the human heart (see Section 7.1), it is likely but not yet published that such heterodimers of D_1_-dopamine receptors and D_2_-dopamine receptors coexist in the human heart. The D_1_-dopamine receptor forms homodimers, but also heterodimers, at least with D_2_-dopamine receptors, even in a myocardial cell line [15,75]. The stimulation of these heterodimeric D_1_-dopamine receptors and D_2_-dopamine receptors can, at least in the brain, potentiate receptor-induced IP_3_-formation. Furthermore, quite a number of other D_1_-dopamine receptor binding proteins have been reported upon [114]. It would be interesting to study these interactions in more detail in the heart.

## 5. Inotropic and Chronotropic Effects of Dopamine in Animal Hearts (Table 2)

There are many studies that have noted the effects of dopamine in the isolated cardiac preparations of animals. They have sometimes focused on atrial functions such as force generation in the left atrial preparations and right atrial preparations, or they have used isolated perfused heart to measure both inotropy and chronotropy. Less often, isolated ventricular cardiomyocytes have been studied. Limited information is available on the age-dependent effects of dopamine under these conditions. One can summarize these studies by saying that usually, dopamine simply acts via β-adrenoceptors, and therefore, the well-recognized positive chronotropic and inotropic effects of dopamine have been reported in nearly all studies (Table 2), with possibly some meaningful exceptions to this general rule (Table 3). All in all, dopamine exerts a positive inotropic effect in isolated electrically stimulated left atrial preparations from guinea pigs, isolated electrically stimulated right papillary muscles from guinea pigs, and isolated electrically stimulated right papillary muscles from cats (Table 2).

### 5.1. Right Atrium

In the isolated blood-perfused canine sinus node preparation, dopamine exerted a positive chronotropic effect, but it was less potent than noradrenaline [30]. The effects of both dopamine and noradrenaline were shifted to the right by prindolol (a β-adrenoceptor antagonist [30]). Whereas the effects of dopamine were not shifted by cocaine, the effects of noradrenaline were shifted to the left by cocaine [30]: this was interpreted as evidence for a direct stimulatory effect of dopamine on β-adrenoceptors [30]. A lower dose of dopamine into the veins of dogs resulted into a positive inotropic effect, and only at a higher dose of dopamine was a positive chronotropic effect noted [30]. It was suggested this might also occur in patients, and thus, dopamine were useful for improving cardiac force generation independent of an undesirable (oxygen demanding) increase in the heart rate [30]. In isolated right atrial canine preparations, dopamine exerted a positive inotropic effect that was blocked by alprenolol, and it was thus mediated by β-adrenoceptors [115]. In isolated rabbit right atrial preparations, a positive inotropic and chronotropic effect of dopamine but not of L-DOPA was likewise reported [116]). The contractile effect of dopamine was attenuated by the reserpine pretreatment of the rabbits [116]. In an isolated blood perfused canine right atrium, dopamine exerted a positive chronotropic effect that was antagonized by propranolol but not by sulpiride (mainly a D_2_- and D_3_-dopamine receptor antagonist: Table 1B), suggesting that the positive chronotropic effects of dopamine in these preparations were β-adrenoceptor-mediated but not D_2_/D_3_-dopamine receptor-mediated. In the atrium of the rat, dopamine exerted a positive inotropic effect that was abrogated by 1 µM propranolol. Therefore, at least in part, the positive inotropic effect was β-adrenoceptor-mediated [117].

### 5.2. Left Atrium

In isolated left cavian preparations, dopamine exerted a positive inotropic effect that was attenuated by propranolol [56]. In isolated cavian preparations, the positive inotropic effect of dopamine was also reported by others; this effect was attenuated by propranolol but not by domperidon, a D_2_-dopamine receptor antagonist (Table 1B [55]). Interestingly, in the same study, fenoldopam (a D_1_-dopamine receptor agonist, Table 1A) or bromocriptine (a D_1,2_-receptor agonist, Table 1A) failed to increase the force of contraction in the isolated electrically driven cavian left atrium [70]. In rabbit atrial preparations, dopamine exerted a positive inotropic effect accompanied by and probably caused by an increase in cAMP levels [67]. These inotropic effects were attenuated by concomitant antagonism on α- and β-adrenoceptors, or when using atrium from reserpine-treated rabbits [67]. These results were explained by a release of noradrenaline by dopamine, and the subsequent stimulation of adrenergic receptors in the rabbit atrium by noradrenaline [67].

### 5.3. Perfused Heart

Dopamine can induce a tachycardia (e.g., isolated rabbits hearts [71]). In the isolated guinea pig heart, dopamine acts mainly via β-adrenoceptors. Dopamine acted to some degree as a direct partial agonist [13], but to the most part as an indirect sympathomimetic agent (releasing noradrenaline), because the inotropic effects of dopamine were shifted to the right (to higher concentrations) after reserpine-pretreatment, but also in the additional presence positive inotropic effect of the β-adrenoceptor-antagonist practolol [13,59,60,118]. In the isolated perfused working heart of the rabbit, dopamine elicited positive inotropic and chronotropic effects, but remarkably, also arrhythmias [70]. In isolated perfused hearts from reserpinized guinea pigs, dopamine raised the beating rate and the force of contraction in a concentration-dependent manner. This effect of dopamine was functionally antagonized by adenosine [119]. Under these conditions, dopamine (1.5 × 10^−8^ mol dopamine as bolus) increased in freeze-clamped hearts the cAMP-content, and this cAMP-content was reduced by concomitantly applied adenosine [119]. In a similar way, adenosine inhibited the dopamine-stimulated activity of adenylyl cyclase activity in broken cell preparations from the guinea pig heart [119]. Interestingly, in the Langendorff-perfused guinea pig heart, dopamine increased the release of noradrenaline from these hearts. This effect was attenuated by a D_1_-dopamine receptor agonist. This might mean that D_1_-dopamine receptors inhibit neuronal noradrenaline release from the guinea pig heart [118].

### 5.4. Papillary Muscles

In the right anterior papillary muscle of dogs, dopamine exerted a positive inotropic effect that was equieffective with noradrenaline, but dopamine was less potent than noradrenaline. The positive inotropic effect of dopamine was greatly attenuated by pindolol (a β-adrenoceptor antagonist), and it was greatly diminished after reserpine-pretreatment of dogs [30]. Dopamine exerts a positive inotropic effect in isolated electrically stimulated left atrial preparations from guinea pigs, isolated electrically stimulated right papillary muscles from guinea pigs, and isolated electrically stimulated right papillary muscles from cats [52]. Like in the dog [30], in isolated feline papillary muscles, dopamine was less potent (pD_2_-value of 5.1) than adrenaline, noradrenaline, or isoprenaline, but it was equieffective with adrenaline and noradrenaline [52,120].

### 5.5. Ventricular Strips

In the guinea pig ventricular muscle strip, dopamine induced a positive inotropic effect through the release of noradrenaline and also due to the direct stimulation of β-adrenoceptors [121]. However, these effects were not antagonized by haloperidol (Table 1B, 3 µM) or phentolamine (an unselective α-adrenoceptor antagonist [121]. Hence, the authors found no evidence for a role of dopamine receptors or α-adrenoceptors under these conditions on force of contraction [121]. An instructive model is the ventricle of the hatched chicken. The ventricle of the hatched chicken has not yet any cardiac innervation: tyramine, an indirect sympathomimetic agent, was without positive inotropic effect [122]. In contrast to tyramine, dopamine exerted a concentration-dependent positive inotropic effect in these ventricles of hatched chicken [122]. As expected, this effect was antagonized by propranolol but not by phentolamine, an α-adrenoceptor antagonist. Moreover, in partially depolarized preparations, dopamine could elicit so-called slow action potentials. This is thought to indicate that dopamine activates L-type Ca^2+^ channels [122]. Interestingly, in the present context, this positive inotropic effect of dopamine was also antagonized by haloperidol (Table 1B, 2 µM). The authors suggested a direct postsynaptic effect of dopamine via β-adrenoceptors, but also via D_2_-dopamine receptors [122]. However, as haloperidol at 2 µM will also block the D_1_-dopamine receptor: the K_i_ values of haloperidol on the D_1_- and D_2_-dopamine receptors indeed overlap. For instance, haloperidol has a pK_i_-value at the D_2_-dopamine receptors of 7.4–8.8, but also pK_i_ values of 7.6–8.2 at the D_1_-dopamine receptors [1]. Hence, the use of haloperidol cannot decide whether D_1_- or D_2_-dopamine or both receptors are involved here. A repetition of this study [122] with better D_1_- and D_2_-dopamine receptor-selective antagonists (Table 1B) might be suggested.

### 5.6. Anaesthetized Animals

Dopamine infusion led to an increase in intraventricular pressure in dogs [56] that was attenuated by verapamil, suggesting that dopamine acts in part by increasing the current through L-type Ca^2+^ channels in the canine ventricle [123]. However, this study could not clarify which receptor is stimulated in the dog by dopamine. In anaesthetized pigs, dopamine also exerted a positive inotropic effect, but these effects were adrenoceptor-mediated and not via dopamine receptors [72].

### 5.7. Cardiomyocytes

Dopamine raised the cAMP-content in isolated rat ventricular cardiomyocytes, but only via β-adrenoceptors [14]. Therefore, it is surprising that dopamine failed to increase contractility in isolated, electrically driven rat adult ventricular cardiomyocytes [62]. However, under these conditions, dopamine decreased isoprenaline-stimulated contractility, and at the same time, it reduced the isoprenaline-stimulated phosphorylation state of phospholamban and troponin I [62]. One might postulate that this cAMP is raised in a compartment that is not involved in contractility. Moreover, the receptor through which dopamine mediated a reduction in isoprenaline-stimulated phosphorylation and contractility was not assessed. One might speculate that the “D_2_-like” receptors are involved because their stimulation would inhibit the activity of adenylyl cyclases, and this decrease is expected to reduce cAMP content in the cell. However, the exact mechanism here also needs further study. The D_1_-dopamine receptor-agonist SKF-38393 (1 µM) raised in 1–4-day-old rabbit cardiomyocytes the current through the L-type Ca^2+^ channel by about 50 %. This increase in current could be attenuated by additionally applied SCH-39390 (Table 1B); the force of contraction was not recorded. The D_1_-dopamine receptor-agonist SKF-38393 (Table 1A) had in these experiments a higher effectivity than dopamine [2]. This is a strong evidence for the functional presence of D_1_-dopamine receptors in the mammalian heart.

### 5.8. Electrophysiological Studies

Early work in multicellular preparations reported on the shortening of the duration of the action potential in rabbit working atrial myocardium, but an increased rate of depolarization in rabbit atrial sinus node cells [116]. The effects were attenuated by pretreatment with reserpine. L-DOPA was without effect [116]. Similarly, a concentration-dependent prolongation, and at later time points, the shortening of the duration of the transmembrane action potential was noted in the isolated guinea pig atrium [124]. In guinea pig ventricular strips in potassium depolarized preparations, evidence for a stimulation by dopamine of the L-type Ca^2+^ channel was reported, but this was antagonized by 10 µM practolol [121]. A direct stimulation of the L-type Ca^2+^ channel in guinea pig ventricular cardiomyocytes was also reported. However, the dopamine was about 100-fold less potent, and about 5-fold less effective than isoprenaline [118]. In sheep Purkinje fibers, that is, in a multicellular preparation, dopamine first shortened then prolonged the action potential duration. The authors concluded that the electrophysiological effects of dopamine were to a large part β-adrenoceptor-mediated [125]. In isolated ventricular cardiomyocytes from rabbit hearts or rat hearts, dopamine increased the current through L-type Ca^2+^ channels [61,118]. This finding is mechanistically important. This finding means: when dopamine under these conditions cannot release noradrenaline from nervous cells (which are absent), dopamine can still stimulate β-adrenoceptors. In this experiment, dopamine would directly stimulate the β-adrenoceptors not via release of neuronal noradrenaline [61,118]. Theoretically, dopamine might still release noradrenaline from the cardiomyocytes themselves. One could test this latter possibility through the use of cocaine.

Another way to examine the possible electrophysiological role of dopamine receptors is to apply known antagonists such as haloperidol, or agonists such as ropinirole (Table 1A). Interestingly, it is widely known that haloperidol can evoke so-called “torsade de pointes” arrhythmias in patients. Fittingly, it was found in isolated rabbit hearts that haloperidol can exert concentration-dependently (a) a negative chronotropic effect, (b) a prolongation of atrioventricular conduction time, (c) prolongation of the action potential duration, and (d) a negative inotropic effect for a concentration of higher than 0.2 µM [126]. This is in accordance with clinical observations that haloperidol prolongs the QTc-interval and slows the heart rate. Thus, in contrast to the inhibition of dopamine receptors by haloperidol, the stimulation of cardiac dopamine receptors, presumably, the D_1_-dopamine receptor and D_2_-dopamine receptor might activate the L-type Ca^2+^ channels and repolarize the K^+^ channels, such as HERG or I(K.r).

In accordance with this idea, it was found that the stimulation of cardiac dopamine receptors with 100 µM dopamine indeed leads to the activation of I(Ca.L) [2], which, however, was more pronounced in newborn than in adult rabbit ventricular cardiomyocytes. This was also shown in HEK293 cells stably expressing I(Ca.L) [127]. Moreover, in rat cardiomyocytes, a block of I(to) channels in both open and open-inactivated states was observed with 1 µM haloperidol [128]. In another study, haloperidol blocked human HERG channels potently with an IC_50_-value of approximately 1 µM [129]. These experimental results are in good accordance to the clinical observation of haloperidol effects and the results in isolated rabbit hearts, and would favor the idea that a stimulation of the dopamine receptor might activate I(Ca.L) and HERG.

However, the situation seems to be even more complex: in canine ventricular cardiomyocytes, the D_2,3_-dopamine receptor agonist ropinirole (Table 1A) concentration-dependently exerts an inhibition of currents through I(Ca.L)-channels and I(K.r)-channels, leading to an action potential prolongation [130]. Four dopamine receptor agonists, namely apomorphine, pergolide, ropinirole, and sumanirole (Table 1A) showed differential effects on human HERG currents, which were blocked by apomorphine, pergolide, and ropinirole, with IC_50_-values of 2.4, 0.12, and 1.2 µM, respectively, and prolonged action potential duration, while sumanirole was widely ineffective [131]. This differential influence on hERG K(+) channel function and cardiac action potential duration by various dopamine receptor agonists might indicate a more complex action, in particular with regard to the HERG and potassium channels, which still needs more patch clamp investigations. In consequence, the effects of dopamine-receptor stimulation in the heart seem to be species- and age-dependent.

### 5.9. Age Dependence of Dopamine-Induced Effects in the Heart

The expression of the D_1_-dopamine receptors (on protein level) fell from the earliest measured value (eight weeks of age) to the age of 20 weeks in rat cardiomyocytes [132]. A similar fall was noted in rabbit hearts on the mRNA and protein-level [2]. There is some evidence that also the function of at least the D_1_-dopamine receptor is age-dependent: a large electrophysiological effect of dopamine in fetal or neonatal rabbit cardiomyocytes faded away (Section 5.8) over time, and thus, in cardiomyocytes from 3–4-month-old rabbits, 1 µM SKF-38393 raised the current by only 15 % [2]. In neonatal canine myocardium, dopamine had a positive inotropic effect. The efficacy of dopamine to increase the force of contraction increased with age and was explained by increasing amounts of releasable noradrenaline as the neonatal canine myocardium ages [133], but this was not affected by haloperidol, and thus was not mediated by haloperidol-sensitive dopamine receptors (that is, D_1_-dopamine receptors and D_2_-dopamine receptors) [133]. In rat atrial preparations, the inotropic effects of dopamine were age-dependent [134]. However, no dopamine antagonists or adrenoceptor antagonists were studied [134]). Thus, it remains unclear as to which receptor is responsible for the age-dependent contractile effect of dopamine in the rat atrium. At certain ages, even a negative inotropic effect of dopamine was recorded in the rat atrium [134]. One might speculate that these negative inotropic effects in rat atrium were D_2_-dopamine receptor-mediated: D_2_-dopamine receptor stimulation is expected to reduce cAMP levels in the mammalian heart (which was regrettably not yet reported). A decrease in cAMP levels would explain a negative inotropic effect to dopamine.

## 6. Effects of Dopamine on Isolated Vessels

Dopamine is assumed to dilate vessels via D_1_-dopamine receptors, through elevation of AMP-levels in these vessels [118]. At least in human coronary arteries [45] and in rat coronary arteries [133], one immunologically detected D_1_-dopamine receptors on smooth muscle cells. D_1_-dopamine receptors have also been detected on vascular endothelial cells [135]: after the stimulation of D_1_-dopamine receptors on these endothelial cells, NO is formed. NO could be released from vascular endothelial cells, and NO would diffuse into the surrounding smooth muscle cells: the smooth muscles relax and this would lead to vasodilatation. D_1_-dopamine receptor-mediated vasodilation was also noted in the canine coronary arteries [11]. In human hearts from organ donors, and therefore probably from non-diseased hearts, one could detect with antibodies the expression of D_1_-dopamine receptors, as well as the other four dopamine receptors with antibodies [136]. D_1_-dopamine receptor knock-out mice (constitutive knock-out) have been generated and exhibit a hypertension that was explained by a lack of the vasodilatory effects of dopamine as the direct consequence of the deletion of D_1_-dopamine receptors from smooth muscle cells in the vessel wall [137,138]. A certain clinical role as a vasodilatory antihypertensive agent is played by fenoldopam, a D_1/5_-dopamine receptor-agonist (Table 1A). Fenoldopam stimulates renal vascular D_1_-dopamine receptors and this is thought to improve renal blood perfusion. If one assumes that “D_2_-like”-receptors reduce cAMP content, one would expect “D_2_-like”-receptors to mediate vasoconstriction. To the best of our knowledge, this “D_2_-like”-mediated vasoconstriction in the coronary arteries and a lack of this vasocontriction in D_2_-dopamine receptor knock-out mice has not yet been reported. Dopamine induced a phenoxybenzamine (an irreversible antagonist at α-adrenoceptors)-sensitive vasoconstriction in isolated human radial arteries [139]. This finding suggests that in human radial arteries, dopamine itself or released noradrenaline stimulated the α-adrenoceptors. Interestingly, there are species differences in the action of dopamine on isolated coronary arterial rings: in canine coronary arterial rings, dopamine leads to relaxation, whereas dopamine leads to vasoconstriction in monkey coronary arterial rings, and notably, also in human coronary arterial rings [140]. These vasoconstrictive effects in monkeys and humans were blocked by phentolamine, an α-adrenoceptor antagonist [140]. One even described some vasodilation due to dopamine in the presence of phentolamine in human coronary arterial rings [140]. These findings suggest that dopamine, or the noradrenaline that dopamine has released, activates vasoconstrictory α-adrenoceptors [140]. If the α-adrenoceptors are blocked, a small vasodilatory action of dopamine can be unveiled [140]. These relaxations were probably not endothelium-mediated because they were still present in rings when the endothelium had been removed [140]. These relaxations, brought about by dopamine, were abolished by SCH23390 (Table 1B), and thus are probably D_1_-mediated [140]. In contrast, the vasodilatory effect of dopamine in the isolated canine artery was abolished by metoprolol and was therefore probably β_1_-adrenoceptor-mediated [140]. The vasoconstrictory effects of dopamine in the human coronary arteries are apparently not region-specific, but they are a general phenomenon in human vessels. In human radial arterial rings (see also above), dopamine likewise led to vasoconstriction [141]. This vasoconstriction was α_1_-adrenoceptor-mediated [141]. The vasoconstriction was more pronounced in the additional presence of the D_1_-receptor antagonist SCH 23390 (Table 1B). These studies suggest that dopamine activates vasodilatory D_1_-dopamine receptors also in the radial artery in humans [140,141]. In these studies, however, cocaine was not added, in order to differentiate whether the stimulation of α_1_-adrenoceptors is mediated by dopamine or noradrenaline, or both. Fenoldopam exerted relaxation in the isolated rings of human saphenous veins. These effects were potentiated by the unselective phosphodiesterase inhibitor, 3-isobutyl-1-methylxanthine, which suggests that this relaxation by fenoldopam is cAMP-mediated via D_1_-dopamine receptors [142].

## 7. The Human Heart

### 7.1. Dopamine Receptors and Their Action in Human Hearts

In human hearts, in principle, dopamine receptors could be detected [136,143,144,145,146]. Specifically, in the human heart, D_1_-dopamine receptors were identified with antibodies in histological slices of the atrium and the ventricle [45]. Likewise, in human atrial and ventricular samples on Western blots with the same antibody, a major band appeared at 55 kDa, and a minor band at 40 kDa [45]. This immunological detection of D_1_-dopamine receptors was confirmed and extended to the detection of not only D_1_-, but also D_2_-, D_4_-, and D_5_- dopamine receptors in the human atrium and left ventricle [143]. However, they could not detect biochemically the D_3_-dopamine receptor in the human heart [143].

In human-induced pluripotent stem-cell-derived cardiomyocytes, D_1_-dopamine receptors and D_2_-dopamine receptors were identified on the mRNA level, and D_1_-dopamine receptors also on the protein level [147]. Thus, there is the possibility of dopamine to increase the force of contraction via the stimulation of D_1_-dopamine receptors on cardiomyocytes in the human heart. Recently, we generated transgenic mice that overexpress the human D_1_-dopamine receptor in the cardiomyocytes only. In these mice, the D_1_-dopamine receptor agonist SKF 38393 exerted a positive inotropic effect. This indicates that the stimulation of the human D_1_-dopamine receptor can, in principle, increase the cardiac force of contraction in the mammalian heart [148]. In isolated human ventricular preparations (from children and adult patients with heart failure equal to or less than New York Heart Association score II), dopamine exerted concentration (0.1–2 mM)-dependent positive inotropic effects that were accompanied by the shortening of the time to peak tension and the time of relaxation, which is typical of cAMP-increasing agents [51]. Dopamine was more effective in human right ventricular preparations, compared to human left ventricular preparations [51]. In all cases, dopamine was less potent and less effective than noradrenaline [51]. The effects of dopamine were abolished by 1 µM bupranolol (a β-adrenoceptor antagonist), suggesting to the authors that the effects of dopamine under these experimental conditions were solely mediated by β-adrenoceptors. Somewhat unexpectedly, dopamine antagonized the positive inotropic effects to noradrenaline in a report [149]. The pD_2_-value was given as 3.5 in ventricular human preparations [149]. In membranes from ventricular human preparations, dopamine stimulated the activity of adenylate cyclase with a similar pD_2_-value of 3.8, which indicated less potent and less effective stimulation than, e.g., noradrenaline under the same experimental conditions [149]. As was the case of contractile responses, dopamine antagonized the effect of isoprenaline to stimulate the activity of adenylate cyclases [149]. Moreover, in isolated human **atrial** preparations, dopamine could induce positive inotropic effects via α_1_-adrenoceptors [49]. However, dopamine failed to mediate positive inotropic effects in isolated **ventricular** human preparations via α_1_-adrenocoeptors [149]. In another study dopamine was more potent and exerted a positive inotropic effect in the human isolated atrium, with a pD_2_-value of 4.5. This positive inotropic effect was both β_1_-adrenoceptor-mediated and β_2_-adrenoceptor-mediated [48]: the positive inotropic effect was antagonized by 300 nM CGP 20712A (a β_1_-adreoceptor antagonist) and 30 nM ICI 118551 (a β_2_-adrenoceptor antagonist, [48]. In the left ventricular papillary muscle from patients with chronic heart failure, dopamine acted likewise via the β_1_-adrenoceptor and β_2_-adrenoceptor, but not via the α_1_-adrenoceptor [52]. In addition, the positive inotropic effects of dopamine were attenuated by cocaine, suggesting that they were, at least in part, mediated by the release of endogenous noradrenaline [52]. It is clear that dopamine stimulates cAMP-generation via adenylyl cyclases in the human heart. In membranes prepared from the left or right ventricles of patients without heart failure, dopamine concentration-dependently increased cAMP production. In the text, the authors write that the effects were neither blocked by haloperidol (10 µM), nor by SCH23390 (10 µM). However, SCH23390 attenuated the effect of dopamine on cAMP-production, which is hard to understand [146]. Moreover, in heart failure, the positive inotropic effect of dopamine is attenuated [52]. In other words, dopamine loses its efficacy. Dopamine has been tried decades ago to treat chronic congestive heart failure. This has basically been unsuccessful, and dopamine has been supplanted by its derivative, dobutamine, which clearly does not act via D_1_-dopamine receptors, but solely via β-adrenoceptors (e.g., [48]). Sometimes, dopamine is given in intensive care units if one wants to increase the force of contraction and to improve renal perfusion, because dopamine via D_1_-dopamine receptors in the kidney can dilate arterial vessels in man. In addition, dopamine seems to reduce splanchnic perfusion. Dopamine is given continuously because of its short half-life time. In cardiogenic or septic shock, high doses of dopamine are needed. At these high doses, dopamine releases noradrenaline in the patient. Noradrenaline then, due to a stimulation of vascular α_1_-adrenoceptors, leads to vasoconstriction. However, when dopamine is used as a vasopressor, frequently, tachyarrhythmic events occur and additional inotropic support is necessary [150]. It is still controversial as to whether the infusion of dopamine will improve or impair the prognosis of patients in intensive care units [151]. In a recent study on patients with postcardiotomy circulatory shock, more patients treated with intravenous dopamine than patients infused with noradrenaline showed tachyarrhythmia [150]. However, the mortality was not increased in those receiving dopamine compared to patients that got noradrenaline [150]. Somewhat paradoxically, dopamine can also induce an acute heart failure (“broken heart” or “Takotsubo syndrome”, [19]). This is usually explained by the acute effects of dopamine in direct or indirect ways to stimulate α_1_-adrenoceptors in coronary arteries, leading to acute cardiac ischemia and dysfunction, but this is still a hypothetical explanation needing experimental confirmation.

There are data that the expression of D_1_-dopamine receptors on mRNA levels increases during human heart failure [80,152]. It is unclear as to whether this upregulation of mRNA is translated into an elevated protein level and an augmented function of the receptor. Indeed, in light of the finding [52] that the inotropic effect of dopamine is reduced in heart failure, it seems likely that the reduced positive inotropic effect of dopamine in heart failure in patients is mainly mediated by the downregulated β_1_-adrenoceptor on which dopamine acts as a direct agonist. It is an interesting hypothesis to test that the attenuated effect of dopamine is in part sustained by upregulated D_1_-dopamine receptors. In other words, one would predict that in failing human ventricular muscle, the efficacy of dopamine were higher than that of isoprenaline. Indeed, data tend to that direction, but the differences were apparently not significant [52]. This argues for more efforts to test this hypothesis.

One has developed an orally active derivate of dopamine called ibopamine (Figure 1), as a positive inotropic drug to treat chronic heart failure. Regrettably, and predictably, in clinical studies, ibopamine increased the mortality mainly due to proarrhythmic effects, a side effect long known for other cAMP-increasing and Ca^2+^-increasing positive inotropic agents [153]. This is not surprising, because one noticed that the active metabolite of ibopamine called epinine (N-methyldopamine) is a pure agonist at the β-adrenoceptors [48,51,154]. From these facts, one can conclude that ibopamine and epinine cannot stimulate human cardiac dopamine receptors, and this cannot be the cause for the failed clinical studies with ibopamine. There are even data indicating that the clinically measured concentrations of epinine in human plasma are too low to even stimulate β-adrenoceptors, because one needs 100- to 1000-fold higher concentrations for the half-maximal stimulation of β-adrenoceptors in isolated human atrial- or ventricular preparations than were reported at the therapeutic dosing of ibopamine in the plasma of patients [154]. Not even on the human vascular dopamine receptor, epinine works as expected: at least in rings from human arteria pulmonalis, epinine did not lead via D_1_-dopamine receptors to a vasodilation, but instead, epinine caused vasoconstriction via α_1_-adrenoceptors [154]. Hence, we would argue that the jury is still out on the usefulness of D_1_-dopamine receptor-stimulating drugs in human heart failure.

Moreover, the effects of dopamine were reduced in efficacy and potency by haloperidol [52]. These are very intriguing findings that may imply that dopamine acts via D_1_-dopamine receptors or the D_2_-dopamine receptors, or both. Recently, we confirmed their data with haloperidol in human atrial preparations, and noted that Odapipam (Table 1B), and to some extent, raclopride (Table 1B) could antagonize the effects of dopamine on the force of contraction in the isolated human atrium (Neumann, Rayo-Abella and Gergs, unpublished observations, 2022). It is therefore possible that the D_1_-dopamine receptor or D_2_-dopamine receptor antagonists (first- or second-generation antipsychotic agents), can have negative inotropic side effects in patients due to antagonistic properties on the cardiac dopamine receptors.

SKF 38393 or fenoldopam prolonged the duration of action potentials, and probably via this prolongation, induced arrhythmias in isolated, spontaneously beating human cardiomyocytes (from stem cells [147]). These effects were attenuated by N-acetylcysteine and aggravated by hydrogen peroxide, suggesting an involvement of free radicals [147]. SCH23390 inhibited the adrenaline-induced prolongation of action potentials, and reduced arrhythmias in isolated spontaneously beating human cardiomyocytes [147]. The prolongation of the action potential was accompanied by and probably caused by an increase in the current through L-type calcium ion currents caused by fenoldopam in isolated spontaneously beating human cardiomyocytes [147]. These effects of D_1,5_-dopamine receptor stimulation have been suggested to contribute to Takotsubo cardiomyopathy [147].

### 7.2. L-DOPA in Human Hearts

In humans, giving L-DOPA led to increasing plasma concentrations of dopamine, and a subsequent positive inotropic effect. However, in subsequent clinical studies, the relevant side effects led to a lack of enthusiasm for this method [155]. However, for lack of good options, L-DOPA was used to some success in children with heart failure [156]. There are clinical observations that in susceptible patients, L-DOPA can cause cardiac arrhythmias, presumably via the formation of dopamine, but possibly also via releases of noradrenaline from cardiac stores [157]. Surprisingly, L-DOPA did not increase the force of contraction in isolated human left ventricular preparations [52]; this suggests that L-DOPA under these conditions is not metabolized to dopamine (Figure 1), which is not easily explained. One might try to measure enzymatically the activity of the DOPA-decarboxylase in human left ventricular papillary muscles to follow this observation further: currently, one might argue that DOPA decarboxylase is not active in the human ventricle. This might be a general phenomenon, as L-DOPA failed to increase the force of contraction in the isolated rabbit atrium, and it did not increase noradrenaline levels in the rabbit atrium [116]. However, in human atrial preparations, we noted a positive inotropic effect of L-DOPA (Rayo-Abella, Gergs, Neumann: unpublished observations 2022). Whether this effect is due to L-DOPA itself or to the production of dopamine from L-DOPA in the human atrium, or the release of noradrenaline, remains an open question. Moreover, L-DOPA has been shown not only to act via the formation of dopamine, but at least in arterial vessels, a specific receptor for L-DOPA has been described upon [158]. It would be interesting to study whether such a receptor is also present in the human heart, and what could be its role in heart failure.

## 8. Dopamine in Disease

### 8.1. Hypertension

Mice with dopamine receptor deletion or mice with dopamine receptor overexpression can develop systemic hypertension. This hypertension can lead to heart failure. In a systematic way, we will subsequently address this issue.

#### 8.1.1. The D_1_-Dopamine Receptor

D_1_-TG, that is, a transgenic mouse with cardiac-specific, inhibitable, overexpression, the D_1_-dopamine receptors has been generated [80]. The mouse has a doxycycline-inhibitable cardiac-specific overexpression, the D_1_-dopamine receptor (“tet-off system”). In these mice, they noted an increased incidence of arrhythmias. To that end, they compared mice, where they stopped to give doxycycline, to mice with continued treatment with doxycycline [80]. They also noticed altered Ca^2+^-transients in neonatal rat cardiomyocytes in culture, that had been infected with an adenovirus coding for the D_1_-dopamine receptors [80]. They noticed spontaneous rises in cytosolic Ca^2+^, and an elevated frequency of Ca^2+^-sparks in these neonatal rat cardiomyocytes with D_1_-dopamine receptor-overexpression. Regrettably, they did not study Ca^2+^-transients in adult mouse cardiomyocytes with D_1_-dopamine receptors overexpression [80]. In contrast, they demonstrated clearly in cardiomyocytes from human cardiac samples using reverse transcription and quantitative polymerase chain reaction that the mRNA for the D_1_-dopamine receptors increased compared to non-failing control samples from humans [80]. Under basal conditions, they noted no enhanced incidence of arrhythmias in mice with a cardiac overexpression of the D_1_-dopamine receptors. In contrast, they could induce arrhythmias with a solution containing dopamine (20 mg/kg) and caffeine (120 mg/kg), supposedly using caffeine as a phosphodiesterase-inhibitor and/or the inhibitor of Ca^2+^-release from the sarcoplasmic reticulum [80]. However, this approach has its pitfalls. Dopamine exerts in mouse isolated electrically paced atrial preparations a positive inotropic effect on its own that is purely mediated by the cardiac β-adrenoceptor, as the positive inotropic effects of 10 µM dopamine could be abrogated with 10 µM propranolol (unpublished observations, Neumann 2021). Interestingly, after aortic banding in mice, the density of β-adrenoceptors also increased as mRNA [80]. The following might have happened: when they infused dopamine, they stimulated both β-adrenoceptors and D_1_-dopamine receptors. Interestingly, they also generated mice with a knock-down of D_1_-dopamine receptors in the mouse heart. In mice with this deletion, they noted a lower incidence of cardiac ventricular arrhythmias after aortic banding compared with WT, if one applied a solution containing dopamine and caffeine. The authors interpreted this as evidence for a proarrhythmic effect of D_1_-dopamine receptor stimulation [80]. Mice with a global complete knock-out of the D_1_-dopamine receptor have been generated [159] that led to hypertension, which should lead over time to heart failure. It is reasonable to assume that hypertension followed the loss of vasodilatory D_1_-receptors in small arterial vessels, which are known to regulate the systemic vascular resistance. However, one can assume that a loss of D_1_-receptors in the kidney may contribute to hypertension, because this loss might alter the sodium metabolism, increase the blood volume, and thereby lead to hypertension. It would be necessary to generate and to study mice with a vessel-specific or renal-specific deletion of the D_1_-receptor to understand the underlying mechanism clearly. However, the animals were apparently not followed for a longer time to assess the development of heart failure. On the other hand, in heart-specific knock-down, one could likewise study mice for a similar period of time. In this way, one could find out whether not only functional renal D_1_-dopamine receptors, but also functional cardiac D_1_-dopamine receptors are crucial to avoid heart failure over time. For instance, one could argue that diminished levels of vasodilatory D_1_-dopamine receptors in mice lead to coronary heart disease. The mortality at aortic banding was higher in mice, which expressed the D_1_-dopamine receptor in cardiomyocytes. They explained the arrhythmias via an increased phosphorylation of the ryanodine receptors in this mouse model [80] such that Ca^2+^ can be released easier from the sarcoplasmic reticulum (Figure 1).

#### 8.1.2. The D_2_-Dopamine-Receptor

Mice with a global deletion of the D_2_-dopamine receptor have been generated and studied [160]. The D_2_-dopamine receptor knock-out mice display hypertension [161] that might lead to cardiac hypertrophy, but this also has apparently not yet been reported. The underlying mechanism must be different from the D_1_-dopamine receptor knock-out. A loss of the D_2_-dopamine receptor in resistance vessels is more likely to lead to vasodilation, and thus, to a lower blood pressure. Hence, other mechanisms must be operational. One hypothesis to test would be that D_2_-dopamine receptor deletion would damage kidney function, leading by a change in blood volume to hypertension. Alternatively, a central mechanism leading to the reduced stimulation of vagal tone, or an increase in sympathomimetic stimulation might be involved. All these suggestions are hypothetical. Likewise, using siRNA, the density of the D_2_-dopamine receptors in only the left kidney could be reduced and this reduction was sufficient to increase blood pressure [162]. In addition, the D_2_-dopamine receptor has been successfully downregulated in a tissue-specific inducible way in mice [163]. Therefore, it should be possible to selectively knock-down the D_2_-dopamine receptor in the heart, in order to differentiate between the renal and cardiac roles of the D_2_-dopamine receptor.

#### 8.1.3. The D_3_-Dopamine Receptor

Mice with a global knock-out of the D_3_-dopamine receptors have been studied [164]. Mice with a global deletion of the D_3_-dopamine receptor display hypertension that is expected to lead to cardiac hypertrophy [165]. However, this was not the case [164]. The mice exhibited a reduced relative heart weight [164]. This was accompanied by histological fibrosis and an increase in the mRNA markers of fibrosis [164]. At the same time, the systolic function of the heart was impaired, which was measured as a reduced left ventricular ejection fraction [164]. The authors speculated they had studied a constitutive knock-out mouse model, and thus, not only renal D_3_-dopamine receptors, but also central D_3_-dopamine receptors and D_3_-dopamine receptors in the nervous cells in the heart were expected to be ablated [164]. This lack of cardiac D_3_-dopamine receptors might have restricted postnatal cardiac growth, accounting for cardiac hypotrophy [164]. Interestingly, the life span of the D_3_-dopamine receptor knock-out mice was shorter than in WT [164]. It was not analyzed as to whether this was a cardiac death, but it would be worthwhile to study. It might be interesting to generate mice with a cardiac-specific knock-out of the D_3_-dopamine receptor, in order to look for a heart-specific effect of the D_3_-dopamine receptor. As a next step, one could induce, e.g., with aortic banding or monocrotaline-treatment heart failure, and see how the mice maintain the cardiac force of contraction. Interestingly, heart failure patients also treated with morphine as an analgesic were more likely to develop cardiac fibrosis [165]. This was also noted after the morphine treatment of mice for one week [165]. When a D_3_-dopamine agonist, pramipexol (Table 1A), was concomitantly applied, morphine-induced cardiac fibrosis was attenuated [165]. This study should probably be repeated with D_3_-dopamine-receptor knock-out mice, as pramipexol (Table 1A) might also act at the D_1_-dopamine receptors (and D_2_-dopamine receptors) in order to understand the underlying mechanisms better [165]. Moreover, it is likely that pramepixol did not act via the D_3_-dopamine-receptors on cardiomyocytes, but those on fibroblasts [166]. Nevertheless, this study might be repeated in patients, with the hope of seeing a clinical effect.

#### 8.1.4. The D_4_-Dopamine-Receptor

Mice with a global knock-out of the D_4_-dopamine receptor have been generated [167]. These mice also develop hypertension, and this leads to cardiac hypertrophy [168]. However, this cardiac hypertrophy was both renally mediated and centrally mediated, but apparently not cardiac-mediated [168]. Here, again the generation of mice with a cardiac-specific knock-out, or at least knock-down would allow for an understanding of the role of the cardiac D_4_-dopamine receptor better.

#### 8.1.5. The D_5_-Dopamine Receptor

Mice with the cardiospecific overexpression of wild type and mutant D_5_-dopamine receptors have been studied [169]. In this case, a global or cardiac-specific overexpression of a mutant of the D_5_-dopamine receptor receptors with a single exchange of amino acids called F173L that showed reduced functionality, displayed cardiac hypertrophy, compared with mice with a cardiac overexpression of the wild type D_2_-dopamine receptor receptors [169]. However, the level of overexpression and the location of the overexpressed protein were not reported [169]. Likewise, mice with a global deletion of the D_5_-dopamine receptor showed cardiac hypertrophy [170]. In the case of mice with a global overexpression of the D_5_-dopamine receptor, the observed hypertrophy is thought to be secondary to hypertension, due to impaired renal function [169]. In contrast, in mice with heart-specific overexpression of the mutant D_5_-dopamine receptor, an increased activity of NAPH oxidase activity was measured and claimed to explain, at least in part, the cardiac hypertrophy measured [169]. A drawback of this study is that the function of wild type mice was not studied for comparison. Moreover, there might be gene dosage effects. In other words, the overexpression might be so high that artificial alterations in the compartmentalization of the D_5_-dopamine receptor might occur, or the D_5_-dopamine receptor might suddenly couple to unphysiological signal transduction pathways. These effects could be tested by making knock-in mice using CRISPR-CAS technology. Mice with a global knock-out of the D_5_-dopamine receptor could be generated [170]. These mice are hypertensive, and probably as a result of hypertension, exhibited increased cardiac weight [170]. However, this hypertension was not mainly renally mediated, but was due to increased sympathetic outflow from the central nervous system [170]. Interestingly, in mice with hypertension and cardiac hypertrophy as a result of aortic banding, the expression of D_5_-dopamine receptors on protein level decreases in the heart [171]. Then the density of D_5_-dopamine receptors was increased via the delivery of the D_5_-dopamine receptor in nanoparticles to the heart; the function of the hearts improved, suggesting to the authors a beneficial role of cardiac D_5_-dopamine receptors, possibly via action on mitochondrial free radical production [169,171]. Of note, these data imply a different regulation of the otherwise similar cardiac D_5_- and D_1_-dopamine receptors: the expression of cardiac D_1_-dopamine receptors increased, but the expression of the D_5_-dopamine receptors decreased in mice after aortic banding [171].

#### 8.1.6. Drug-Induced Hypertension and Dopamine Receptors

Monocrotaline is a naturally occurring alkaloid. Monocrotaline damages endothelial cells, for instance, in lung pulmonary arteries. This can lead in humans and laboratory animals to pulmonary hypertension, and firstly, right-sided heart failure that can aggravate to global heart failure [63,172]. Giving monocrotaline to rats for some time, and then studying the isolated perfused heart (Langendorff-preparation) of monocrotaline-pretreated rats (but not in untreated control rats), dopamine induced a positive inotropic effect that was blocked by SCH-23390 (10 μM, Table 1B). Thus, under pathophysiological conditions (here: heart failure), a functional cardiac D_1_-dopamine receptor is unveiled [63]. Thus, in principle, D_1_-dopamine receptors in the heart of the adult rat can be directly stimulated by dopamine, leading to a positive inotropic effect. However, even under these conditions, the inotropic effect was quite small (about 15 % increase in the force of contraction [63]). Nevertheless, this positive inotropic effect via the D_1_-dopamine receptors might present a protective mechanism when the cardiac inotropic function is compromised, to ensure that cardiac force can still be raised to meet the necessities of the body. From these experimental data, one can hypothesize also that the known upregulation of the mRNA of the D_1_-dopamine receptor in human heart failure [80] might serve to sustain force generation in human heart failure.

#### 8.1.7. Genetically Induced Hypertension

In spontaneously hypertensive rats, a reduced activity of β-hydroxylase was noted in the heart ventricle, compared to normotensive control rats [173]. It was speculated that this defective degradation of dopamine might contribute to the genesis of hypertension [173]. This model has apparently not been studied further, and therefore, the underlying mechanisms remain enigmatic.

### 8.2. Ischemia

During ischemia and hypoxia, dopamine can be released in the neuronal cells (e.g., [174,175]), and thus, high dopamine levels can be reached. If this mechanism also occurred in the heart, these ischemia-induced dopamine elevations might be able to stimulate D_1_-dopamine receptors, and thus they might induce arrhythmias. Fittingly, dopamine is known to induce cardiac arrhythmias in general [70,80,144], but especially in cardiac ischemia [176,177].

Simulated ischemia and reperfusion led in isolated neonatal rat cardiomyocytes to an increased expression (on protein level) of D_1_-dopamine receptors [78], but also a heightened expression of D_2_-dopamine receptor, which led to impaired mitochondrial function [78]. This might imply a certain relevance of D_1_-dopamine receptors in patients with myocardial infarction. Oxidative stress can reduce at least the mRNA for the D_1_-dopamine receptor [110]. The underlying mechanism may lie in the observation that oxidative stress blocks the activity of the transcription factors AP1 and SP3, and thereby reduces D_1_-dopamine receptor expression [110]. Hypoxia can raise the expression of D_1_-dopamine receptors (with Western blotting) in cultivated neonatal rat cardiomyocytes [78]. Blocking the D_2_-dopamine receptor by raclopride (Table 1B) in neonatal rat cardiomyocytes induced autophagy in these cells [178], likewise suggesting a role of D_2_-dopamine receptors in ischemia and hypoxia. Moreover, endothelin or angiotensin II-induced hypertrophy in isolated cultured rat neonatal cardiomyocytes was attenuated by bromocriptine (Table 1A), suggesting that the D_2_-dopamine (or D_1_-dopamine receptor) might be involved in cardiac hypertrophy.

### 8.3. Genetically Induced Heart Failure

Interestingly, in a genetic model of cardiomyopathy, the cardiomyopathic hamster, when the cardiomyopathy progressed, a decrease in the activity of the β-hydroxylase activity in the heart was reported. Hence, an increase in cardiac dopamine levels seems to correlate in this model with heart failure [179].

### 8.4. Sepsis

In experimental sepsis (the injection of lipopolysaccharide, a well-established model of sepsis), the blood–brain barrier may open, which is detrimental. The stimulation of the D_2_-dopamine receptors using the agonist cabergoline (Table 1A, 20 µM), possibly by closing the tight junctions, improves the barrier function under these conditions [180]. This might result from a stimulation of the D_2_-dopamine receptors on the endothelial cells in the brain. However, cabergoline is a very promiscuous drug: it is an agonist at various serotonin receptors, an antagonist at adrenergic receptors, and it has a pKi of 6.7 at the D_1_-dopamine receptors [1,181]. Hence, one could argue that the D_1_-dopamine receptor was involved, as 20 µM cabergoline would effectively also stimulate the D_1_-dopamine receptor. Hence, studies with more selective agonists or genetically manipulated mice are necessary, to better understand the role of dopamine receptors in sepsis.

One could regard malaria as an infectious disease possibly leading to sepsis. In the brains of mice with experimental malaria, the expression of D_1_-dopamine receptors (and D_2_-dopamine receptors) was enhanced on a protein level. Fittingly, the phosphorylation state of DARPP32, the phosphorylation state of the cAMP-dependent protein kinase (Figure 2), and the expression of PLC (Figure 1) in the brains of these mice were found to be elevated [182], suggesting that infections can affect the expression and function of dopamine receptors. The hearts of these animals were regrettably not studied, which might have answered the question as to what role dopamine receptors in the heart play in cardiac inflammation, specifically malaria-induced inflammation.

The stimulation of the D_5_-dopamine receptor could alleviate the symptoms of bacterial experimental sepsis in mice [183]. This was explained on a molecular base in the following way. D_5_-dopamine receptors form a complex involving PP2A (Figure 2) that inhibits the proinflammatory pathway that is activated via TLR2-receptors in leucocytes [183]. However, looking closely at their data, infection also increased the expression of the D_1_-dopamine receptor, but less than the expression of the D_5_-dopamine receptors [183]. However, as noted above, the D_1_- and D_5_-dopamine receptors belong the same family of dopamine receptors. Thus, these data support a role of dopamine receptors in cardiac inflammation, and they might indicate a clinically useful beneficial role of “D_1_-like” receptors against myocarditis.

In experimental sepsis (through intestinal puncture) in rats, dopamine turned out to be less potent and effective than the pure β-adrenoceptor agonist dobutamine, compared with rats without sepsis [184]. Here, the problem is obvious that the effect of dopamine is most probably via the adrenergic receptors, and a role of the cardiac dopamine receptors remains hypothetical, as dopamine in the rat heart would preferentially, if not exclusively, act on the adrenergic receptors (Table 2).

## 9. Clinical Relevance

As always, it should be kept in mind that overexpression and knock-out mouse models are helpful in understanding, for instance, the role of dopamine receptors. However, there are species differences between mice and humans. Some receptors are functionally absent in mice, and their cardiac knock-out will thus not lead further. Moreover, one problem with overexpression and the knock-out of receptors resides in the observation that this may lead in the heart to the activation of artificial signal transduction pathways. Thus, while genetically manipulated mice have been very important for understanding mechanisms, one has to be careful when translating these findings into the clinic, and one has to avoid pitfalls. D_1_-dopamine receptors in the brain (but as an unintended side effect, also in the heart) can be blocked by many antipsychotic drugs ([166], Table 1B). Interestingly, most antipsychotic drugs also block D_2_-dopamine receptors [185,186] (Table 1B). In patients with heart failure, the expression of D_1_-dopamine receptors [80] and of the D_5_-dopamine receptor in the heart was increased [170]. This might be a compensatory mechanism (see above).

Lower concentrations of dopamine could theoretically dilate the coronary vessels via D_1_-dopamine receptors or β-adrenoceptors, but dopamine also induces vasoconstriction in isolated human coronary rings, probably via α_1_-adrenoceptors [140]. Moreover, in the human heart, dopamine could exert positive inotropic cardiac effects via the stimulation of α_1_-adrenoceptors, D_1_-dopamine receptors, or β-adrenoceptors [39]. Higher concentrations of dopamine in clinical situations would release noradrenaline: thus, dopamine acts as an indirect sympathomimetic drug and the released noradrenaline stimulates vasoconstriction via α_1_-adrenoceptors. As mentioned above, the published data are somewhat controversial. Even nowadays, dopamine is used in the clinic to raise blood pressure, for instance, in patients with sepsis [39]. There are data in patients that noted after intravenous infusion a direct positive inotropic effect of fenoldopam (a D_1_-dopamine receptor agonist) or propylbutyldopamine (a D_2_-dopamine receptor agonist), suggesting that the D_1_-dopamine receptor and the D_2_-dopamine receptor in the human heart can directly or indirectly increase the force of contraction [187].

A Japanese group convincingly reported on an increased cardiac expression of the mRNA for the D_1_-dopamine receptors in patients with heart failure, compared to the matched controls [80]. However, the protein data were lacking [80]. Moreover, these data would be expected to lead to a more effective positive inotropic effect of D_1_-dopamine receptor simulation, compared to β-adrenergic stimulation. Data in this regard are also not yet convincing.

All dopamine agonists clinically used to treat Morbus Parkinson, besides the intended agonistic effects on D_2_-dopamine receptors, also have agonistic effects on D_1_-dopamine receptors [39,185] (see Table 1A for examples). If one gives patients L-DOPA without also supplying a decarboxylase-inhibitor, then one actually treats patients with dopamine because L-DOPA is quickly converted to dopamine in the periphery. D_2_-dopamine receptor agonists such as bromocriptine or pergolide have a higher potency on D_2_-dopamine receptors than D_1_-dopamine receptors. However, pergolide and bromocriptine have an affinity of 0.1 µM (as Ki) or 0.6 µM at D_1_-dopamine receptors; therefore, they can act as agonists at the D_1_-dopamine receptors [10] (Table 1A). Interestingly, dopamine appears to have antioxidant effects that were utilized in patients undergoing cardiac transplantation [188]. Indeed, when prospective heart donors were treated with an infusion of a low-dose dopamine compared to non-treated control donors, this dopamine infusion was accompanied by a better clinical outcome in those patients that received the transplanted heart [188]. The underlying mechanism is speculative, but the beneficial stimulation of cardiac dopamine receptors is an attractive hypothesis that could be tested.

## 10. Outlook

There is growing evidence that dopamine cannot only act on the contractile function of the heart, especially the human heart, by directly or indirectly stimulating α- and β-adrenoceptors. In addition, dopamine seems to stimulate the D_1,5_-dopamine receptor in the sinus node, the atrium, and the ventricle of the human heart (or at least, in the mammalian heart). Genetic models in mice have been helpful in this regard, and new genetic models might in many ways add to our knowledge of the cardiac function and the underlying signal transduction mechanisms in the human heart. These mice models might be used in subsequent work to obtain models for cardiovascular diseases such as arrhythmia, genetically induced heart failure, septic heart failure, and drug-induced heart failure. Studying these mechanism, it should be possible to repurpose known agonists and antagonists at the D_1,5_-dopamine receptors, or to invent new drugs that are biased agonists at relevant dopamine receptors, or to inhibit deleterious signal transduction pathways. More effort might be put into the study of “D_2_-like” receptors in the heart, where only very scarce data seem to be available.

## 11. Summary

In summary, this review tried to convince the reader that the study of dopamine in the heart, and a fresh look at cardiac dopamine receptors, especially D_1_-dopamine receptors, is a timely and promising endeavor in experimental and clinical cardiovascular pharmacology. We hope that these efforts will improve patient care, and possibly reduce cardiovascular morbidity and mortality in the years to come.

## Figures and Tables

**Table 1:** (**A**) Dopamine receptor agonists. The main dopamine receptors on which the commonly used dopamine receptor agonist acts are listed here in five columns. Dopamine is an agonist at all dopamine receptors, albeit with varying affinities. One would predict in the heart that first high-affinity dopamine receptors, and thereafter, low-affinity receptors, will be stimulated by dopamine. Note that there is no drug currently available that allows for distinguishing between D_1_-dopamine and D_5_-dopamine receptors. Usually, antagonists do not distinguish between D_2_-, D_3_-, D_4_-dopamine receptors. Moreover, at higher concentrations, for instance, fluphenazine antagonizes also at the D_1_-dopamine receptors. Moreover, antagonists usually are not dopamine receptor-selective. For instance, metoclopramide also acts antagonistically on cardiac 5-HT_4_-serotonin receptors and domperidon on cardiac potassium channels. (**B**) Dopamine receptor antagonists. The main dopamine receptors on which commonly used dopamine receptor antagonists act are listed here in five columns. Note that there is no antagonist that is currently available that allows to distinguish between D_1_-dopamine and D_5_-dopamine receptors. Usually, antagonists do not distinguish between D_2_-, D_3_-, D_4_-dopamine receptors. Moreover, at higher concentrations, for instance, fluphenazine antagonizes also at D_1_-dopamine receptors. Moreover, antagonists usually are not dopamine receptor-selective. For instance, metoclopramide also acts antagonistically on cardiac 5-HT_4_-serotonin receptors, and domperidon acts antagonistically on cardiac potassium channels. Superscript numbers indicate which drug was used in which reference. Ki indicates the half maximum concentration to inhibit a function and pKi represents its negative decadic logarithm.

**(A) Dopamine Receptor Agonists**					
**D_1_**	**D_2_**	**D_3_**	**D_4_**	**D_5_**	**Reference**
dopamine pK_i_: 4.3–5.6	dopamine pK_i_: 4.3–5.6	dopamine pK_i_: 6.3–7.4	dopamine pK_i_: 7.6	dopamine pK_i_: 6.6	Myslivecek 2022 [1]
^1^ SKF-38393	^2^ MLS1547			^1^ SKF-38393	^1^ Ding et al. 2008 [2] ^2^ Myslivecek 2022 [1]
AT77636	rotigotine	rotigotine	rotigotine	AT77636	Myslivecek 2022 [1]
^1^SKF-81297	^1^ ropinirole	^1^ ropinirole	^2^ quinpirole	^1^ SKF-81297	^1^ Myslivecek 2022 [1] ^2^ Yamaguchi et al., 1997 [3]
^1^ SKF-83959	^1,2^ pramipexol	^1,2^ pramipexol	^2^ pramipexol	^1^ SKF-83959	^1^ Myslivecek 2022 [1] ^2^ Piercey et al., 1996 [4]
fenoldopam (=SKF 82526)	^2^ PD 128907	PD 128907		fenoldopam (=SKF 82526)	Myslivecek 2022 [1]
^2^A68930	^1^ PD16077		^1^PD16077		^1^ Myslivecek 2022 [1] ^2^ DeNinno et al., 1990 [5]
^2^ chloro APB	^1^ A412997	^1^A412997	^1^ A412997		^1^ Myslivecek 2022 [1] ^2^ Neumeyer et al., 1991 [6]
dihydrexidine					Salmi et al., 2004 [7]
	apomorphine				Myslivecek 2022 [1]
	propylbutyl-dopamine				Zhao et al. 1990 [8]
	SKF81297				Rashid et al., 2007 [9]
	quinpirole				Rashid et al., 2007 [9]
bromocriptine 0.1 µM (as Ki)	bromocriptine				Kvernmo et al., 2008 [10]
pergolide 0.6 µM (as Ki)	pergolide				Kvernmo et al., 2008 [10]
**(B) Dopamine Receptor Antagonists**					
**D_1_**	**D_2_**	**D_3_**	**D_4_**	**D_5_**	**Reference**
^2^ haloperidol	^2^ haloperidol	^2^ S33084	^2^ L745870		^2^ Myslivecek 2022 [1]
^1^ SCH-38390	^2^ pipotiazine	^2^ SB277011-A	^2^ sonepiprazole	^1^ SCH-39390	^1^ Ding et al. 2008 [2]
^2,3^ SKF-83566	^2^ perospirone	^2^ perospirone	^2^ perospirone	^2^ SKF-83566	^2^ Myslivecek 2022 [1] ^3^ Kopia and Valocik 1989 [11]
^2^ ecopipam	^2^ raclopride	^2^ raclopride	^2^ A-381393	^2^ ecopipam	^2^ Myslivecek 2022 [1]
^2^ SCH23982	^2^ ML321	^2^ sulpiride	^2^ sulpiride		^2^ Myslivecek 2022 [1]
^1^ odapipam (=NNC 756 Ki: 0.17 nM)	^1^ odapipam (Ki: 942 nM) ^2^ prochlorperazine	^2^ prochlorperazine	^2^ prochlorper-azine		^1^ Andersen et al., 1992 [12] ^2^ Myslivecek 2022 [1]
	NGB 2904	NGB 2904	NGB 2904		Myslivecek 2022 [1]
	metoclopramide	S14297	S14297		Myslivecek 2022 [1]
	domperidon				Myslivecek 2022 [1]
	pimozide	pimozide	pimozide		Lumley et al., 1977 [13]
fluphenazine	fluphenazine				Vettel et al., 2014 [14]
	melperone				Lencesova et al., 2017 [15]
cabergoline	cabergoline				Myslivecek 2022 [1]

Pergolide and bromocriptine have an affinity of 0.1 µM (as Ki) or 0.6 µM at D_1_-dopamine receptors; therefore, they can act as agonists at D_1_-dopamine receptors.

**Table 2:** Dopamine actions at non-dopamine receptors in the heart. Here, receptors other than dopamine receptors are put together, on which dopamine can act in the heart as an agonist. This table is intended to remind readers that dopamine is not selective agonist at dopamine receptors, but it shows some promiscuity. The first row reports the affinity of dopamine as these non-dopamine receptors. The message here is that only rarely are dopamine receptors involved in the cardiac actions of dopamine. Usually, the release of noradrenaline is involved, and this noradrenaline then stimulates adrenergic receptors in the heart. Sometimes, in addition, dopamine itself can stimulate adrenergic receptors. In some cases, it is concentration-dependent for which receptor in the heart dopamine acts. Note that there are huge species differences, and no species has been described where the cardiac effects of dopamine are solely or even mainly dopamine receptor-mediated. Abbreviations used: LTCC: L-type calcium ion current. PIE: positive inotropic effect. PCE: positive chronotropic effect. Columns refer to the receptors listed in the first row. LTCC: L-type calcium ion current, n.d.: not determined. Superscript numbers indicate which drug was used in which reference.

Receptor	α_1_-Adrenergic pKi: 5.6	β_1_-Adrenergic pKi: 5.0	β_2_-Adrenergic pKi: 4.3	Other Effects	Reference Myslivecek 2022 [1]
Human right atrium	^2^	^1,4^	^1,4^	^3^ D_1_-induced release of noradrenaline	^1^ Deighton et al., 1992 [48] ^2^ Wagner et al., 1980 [49] ^3^ Rump et al., 1995 [50] ^4^ Bravo et al., 1991 [51]
Human ventricle	No ^1^	^1,3,4^	^1,3,4^	release of noradrenaline ^1,2^ ^1^ D_1_- or D_2_-mediated	^1^ Brown et al., 1985 [52], ^2^ Port et al., 1990 [53] ^3^ Brown et al., 1985 [52] ^4^ Bravo et al., 1991 [51]
Cat right papillary muscle				^1^ Receptor type not studied	^1^ Brown und Erdmann 1985 [54]
Guinea pig right atrium		^1,2^ propranolol	^1,2^ propranolol	^1,3,4^ PCE attenuated by reserpine ^4^ inhibition of noradrenaline synthesis reduced potency of dopamine, ^4^ poteniated by cocaine, ^5^ not attenuated by pimozide	^1^ Martinez-Mir et al., 1987 [55] ^2^ Einstein and Barrett [56] ^3^ Tsai et al., 1967 [57] ^4^ Brown 1990 [58] ^5^ Lumley et al., 1977 [13]
Guinea pig left atrium		^2^ propranolol ^4^	^2^ propranolol ^4^	^1^ receptor type not studied, ^2^ attenuated by reserpine, ^3^ not attenuated by haloperidol, ^4^ inhibition of noradrenaline synthesis reduced potency of dopamine, ^4^ MAO-Inhibition increased potency of dopamine, ^4^ not altered by SCH23390, domperidone, cocaine	^1^ Brown and Erdmann 1985 [54] ^2^ Martinez-Mir et al., 1987 [55] ^3^ Einstein and Barrett [56] ^4^ Brown 1990 [59]
Guinea pig right papillary muscle	not involved			Receptor type not studied, attenuated by reserpine, not attenuated by haloperidol, inhibition of noradrenaline synthesis reduced potency of dopamine, MAO-inhibition increased potency of dopamine	Brown and Erdmann 1985 [54]
Isolated guinea pig heart				^1,2^ noradrenaline release, ^2^ PCE	^1^ Lumley et al., 1977 [13] ^2^ Habuchi et al., 1997 [60]
Guinea pig right atrial cardiomyocytes				LTCC increases	Habuchi et al., 1997 [60]
Rat neonatal cardiomyocytes		atenolol		increase in cAMP	Vettel et al., 2014 [14]
Rat atrium					Zhao et al., 1997 [61]
Rat ventricular cardiomyocyte		^1,2^ isoprenaline effect is attenuated by dopamine		^2^ no PIE of dopamine	^1^ Zhao et al., 1997 [61] ^2^ Shi et al., 2017 [62]
Isolated rat heart monocrotaline treated				PIE was SCH23390-sensitive	Piao et al., 2012 [63]
Dog		^1,2^ propranolol ^3^ practolol	^1,2^ propranolol	^1,3^ PCE, ^2,3^ PIE	^1^ James et al., 1970 [64] ^2^ Black and Rolett 1968 [65] ^3^ Lumley et al., 1977 [13]
Rabbit left atrium		^1,2^ pindolol	^1,2^ pindolol	^2^ PIE attenuated by cocaine and reserpine	^1^ Endoh et al., 1976 [66], ^2^ Brodde et al., 1980 [67]
Rabbit right ventricular papillary muscle	^1,2,3^	^1,2^ pindolol	^1,2^ pindolol	^2^ PIE attenuated by cocaine, ^2^No effect of pimozide	^1^ Schümann et al., 1977 [68] ^2^ Brodde et al., 1980 [67] Motomura et al., 1978 [69]
Rabbit isolated ventricle				Noradrenaline release	Wakita 2007 [70]
Rabbit ventricular cardiomyocyte			LTCC	SCH23390-sensitive LTCC	Ding et al., 2008 [2]
Isolated rabbit hearts					Vigholt Sørensen et al., 1986 [71]
Anesthetized pig				PIE not D_1_ mediated	Van Woerkens et al., 1991 [72]

**Table 3:** Species-dependent functional actions of dopamine via dopamine receptors on myocardial tissue. Here, the meagre evidence is assembled where dopamine not exclusively but measurably exerts functional cardiac effects via dopamine receptors. Abbreviations: LTCC: L-type calcium ion current. Moreover, we mention which agonist or antagonist for dopamine receptors was used to reach a conclusion. Please see Table 1A,B for the appropriateness of the drugs used in these studies.

Tissue/Species	LTCC	Agonist	Antagonist	Force	
Neonatal rabbit cardiomyocyte	Large increase	SKF-38393, dopamine	SCH-39390	n.d.	Ding et al., 2008 [2]
Adult rabbit cardiomyocyte	Small increase	SKF-38393, dopamine	SCH-39390	n.d.	Ding et al., 2008 [2]
Isolated heart from monocrotaline- treated rat	n.d.	dopamine	SCH-39390	small increase	Piao et al., 2012 [63]

**Table 4:** Further dopamine binding receptors or other proteins: Dopamine does not only act on cardiac adrenergic receptors, as seen in Table 2, but dopamine can inhibit the transporter listed here or activate the receptors listed here.

	pKi:	Reference
α_2_-adrenoceptor	6.01	Myslivecek 2022 [1]
DAT: dopamine transporter	5.3	Myslivecek 2022 [1]
NET: noradrenaline transporter	4.55	Myslivecek 2022 [1]
SERT: serotonin transporter	4.53	Myslivecek 2022 [1]
Melatonin receptors MT_1A_	5.15	Myslivecek 2022 [1]
Melatonin receptors MT_1B_	5.04	Myslivecek 2022 [1]
TAAR: trace amine associated receptors	6.38	Borowsky et al., 2001 [73]

## Data Availability

All reasonable data requests will be answered by the corresponding author.
